# On the Electronic Nature of Open-Shell Germylene Complexes of M^I^ Cations (M = Fe, Co, Ni)

**DOI:** 10.1021/acs.inorgchem.6c01906

**Published:** 2026-06-25

**Authors:** Annika Schulz, Jonas L. Gilch, Konstantin B. Krause, Christian Limberg, Terrance J. Hadlington

**Affiliations:** † Fakultät für Chemie, School of Natural Sciences, TU München, Lichtenberg Strasse 4, 85749 Garching, Germany; ‡ Institut für Chemie, 9373 Humboldt-Universität Zu Berlin, Brook-Taylor-Strasse 2, 12489 Berlin, Germany

## Abstract

While the coordination chemistry of heavier tetrylene ligands is generally well established, their effect on the electronic nature of open-shell metal centers is underexplored. This work takes steps to define these effects in an analogous series of cationic germylene-M^I^ complexes (M=Fe, Co, Ni). First, the electronic ground-states of toluene-coordinated M^I^ synthons, [IPr·M­(η^6^-tol)]^+^ (IPr = [{(H)­CN­(Dip)}_2_C:]; Dip = 2,6-C_6_H_3_) are elucidated using CAS-SCF calculations, defining a rare non-Aufbau for the Fe­(I) derivative. Following this, a combination of experimental and computational investigations on novel paramagnetic germylene complexes **2** (Fe), **3** (Co), and **4** (Ni) uncover a doubly quasi-degenerate iron­(I) complex, with additional low-energy excited states for Fe (∼0.1 eV) and Co (∼0.2 eV), despite the highly unsymmetrical ligand sphere. In all cases significant spin-polarization on the ligand′s Ge center is found, which lends these systems higher than expected magnetic moments, correlating with known spin–orbit effects for the heavier *p*-block elements. This study thus gives new insights into the nature of electronic communication between heavier tetrylenes and open-shell 3d-metal centers.

## Graphical abstract

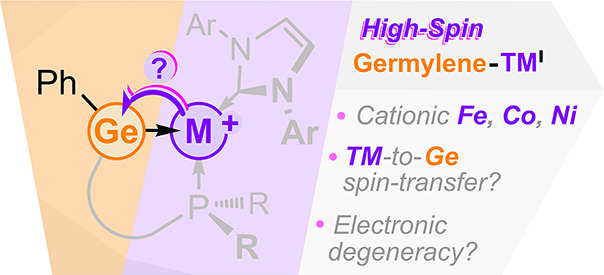

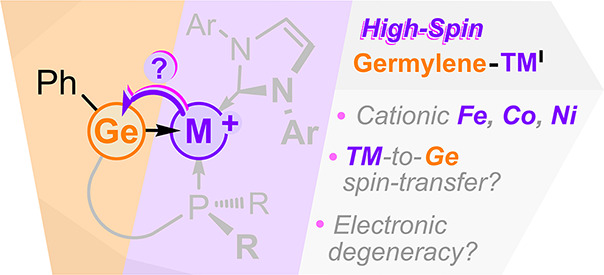

## Introduction

The isolation of the first stable carbene transition-metal (TM) complex by E. O. Fischer in the 1960s marked a landmark discovery in modern organometallic chemistry.^1^ Since then, a vast number of TM complexes stabilized by carbene ligands have been reported, with applications ranging from catalysis to functional materials and biomedical chemistry.^2–6^ In recent years, attention has increasingly encompassed the heavier group 14 analogues, *viz*. tetrylenes, which feature E centers in the +II oxidation state (E=Si, Ge, Sn, Pb).^7^ Heavier tetrylenes exhibit distinct electronic features when compared with carbenes: they are increasingly stable in a lower oxidation state due to higher HOMO–LUMO separation on descending group 14.^8,9^ Concomitantly, their decreasing electronegativity renders the heavier group 14 center, E, more Lewis acidic than C in carbenes. Their bonding interactions with a metal are thus defined by dative donation and back-donation (Figure 1 **A**). These properties can be exploited to move beyond the classical role of carbenes as spectator ligands, which are often solely used to stabilize reactive metal centers, and instead enable tetrylenes to act as noninnocent ligands; bearing Lewis acidic and Lewis basic properties opens opportunities for cooperative reactivity involving both the TM and tetrylene centers.^7,10–13^ For this, an in-depth understanding of the electronic nature of both E and TM centers in such processes is of significant importance.

**Figure 1:**
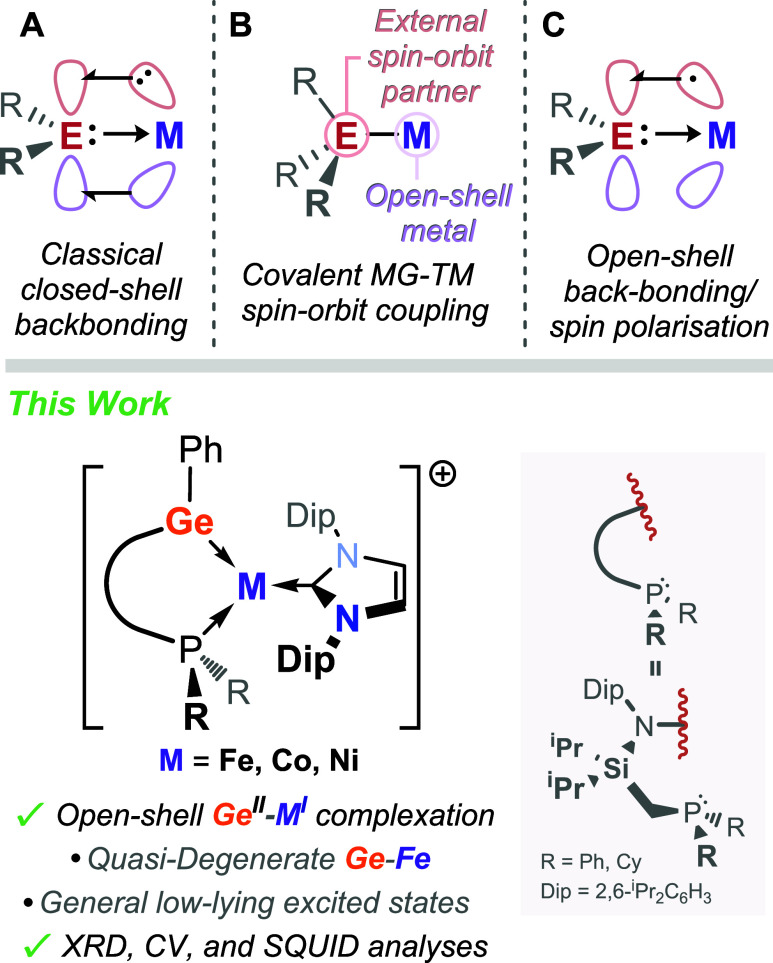
(A): classical back-donation in transition-metal tetrylene complexes; (B): known effects of heavier tetryl elements in affecting spin–orbit coupling in transition-metal systems; (C): hypothesized spin delocalization/polarization in open-shell transition-metal tetrylenes complexes; *below*: a summary of the systems reported in this work.

In this regard, broader coordination chemistry has shifted toward the investigation of systems involving 3*d*-metals, with the need to move away from catalysts which rely heavily on expensive and scarce metals such as platinum, palladium, ruthenium, or osmium.^14–16^ Over the past decade, numerous iron-, cobalt-, and nickel-based complexes have proven to be highly active in, for example, hydrogenation,^17,18^ hydroformylation,^19^ and hydrosilylation processes.^20,21^ Still, the heavier tetrylene coordination chemistry of these metals is relatively underexplored - this is particularly true for open-shell spin states, *e*.*g*. to the best of our knowledge base-free tetrylene complexes of Fe^I^ and Ni^I^ are unknown,^7^ outside of our own work.^22^ An intriguing point here regards the classification of heavier tetrylenes within established ligand field theory, i.e. as strong- or weak-field ligands, and the effect of their inherent ambiphilicity on the ligand field. Szilvási and co-workers have computationally shown that silylenes and germylenes can reach the σ-donor capacity of N-heterocyclic carbenes, while demonstrating a greater π-acceptor capacity.^23,24^ More broadly, heavier tetryl groups are known to increase spin–orbital coupling and magnetic anisotropy through covalent bonding to open-shell TM centers (Figure 1B).^25,26^ This ability to tune magnetic anisotropy has notable implications in catalysis and molecular magnetism.^27–31^ Given the vast field of heavier tetrylene chemistry, an understanding of such effects in *dative* tetrylene-TM systems may open new possibilities in molecular spin-tuning (Figure 1C).

In order to expand our current understanding of the electronic nature of open-shell 3d-metal tetrylene complexes, herein we expand on known examples of such species in the synthesis and isolation of an analogous series of high-spin iron­(I), cobalt­(I), and nickel­(I) complexes, bearing a chelating germylene framework. These cationic complexes are investigated regarding their magnetic properties, both experimentally and computationally, giving important insights into this underdeveloped subclass of coordination chemistry.

## Results and Discussion

We have earlier demonstrated that [IPr·M­(η^6^-tol)]­[BAr_4_^F^] (M=Fe, Co, Ni; IPr = [(H)­CN­(Dip)_2_C:]) complexes can serve as efficient [NHC·M]^+^ transfer reagents.^32^ Building on this, here we extend this strategy to the germylenes ^R^L­(Ph)­Ge: (**1a**, R = Ph; **1b**, R = Cy; ^R^L = [{R_2_PCH_2_Si­(^i^Pr)_2_}­(Dip)­N]^−^). In each case, the reaction furnished intensely colored oils corresponding to compounds **2** (Fe), **3** (Co), and **4** (Ni), which could be crystallized from fluorobenzene solutions layered with pentane in yields between 75 and 85% (Scheme 1). All compounds are ^31^P­{^1^H} NMR silent, and broad signals over a wide chemical shift range are observed in ^1^H NMR spectra, indicative of paramagnetism (*vide infra*). The molecular structures of all novel complexes have been attained via X-ray crystallography (*e*.*g*. Figure 2), confirming the connectivity in these complexes. We note that X-ray crystal data for **3a** was previously reported.^33^ These data indicate three-coordinate TM centers for all systems - a Y-shaped geometry is apparent for Fe and Co, while a deviation toward T-shaped is observed for Ni (i.e. ∠_PNiC_ = 154.2(1)°; ∠_GeNiC_ = 112.4(1)°). The former likely arises from the chelating ligand bite angle, ranging from 88.43(2)° (Fe) to 91.99(3)° (Ni), while the deviation toward T-shaped may indicate an in-plane SOMO (singularly occupied molecular orbital; *vide infra*), based on earlier reported examples of T-shaped Ni systems.^34,35^ All systems demonstrate long Ge-M bonds (e.g. **2**: d_GeFe_ = 2.3542(9) Å; **3b**: d_GeCo_ = 2.324(1) Å; **4a**: d_GeNi_ = 2.3082(7) Å), indicative of high-spin metal centers. Comparing, for example, the Ge–Ni bond in Ni^I^ complex with that in our previously reported neutral Ni^0^ congener, ^R^L­(Ph)­Ge·Ni·IPr, we see a Ge–Ni distance of 2.219(1) Å. A similar comparison can be made for Fe, using our previously reported closed-shell cationic germylene-iron(0) complex [^R^LGe·Fe·IPr]^+^.^22^ In that complex, a short Ge–Fe bond of 2.198(1) Å is found, marking an elongation in **2** of ∼0.16 Å. This aligns with the expansion of the metal radius in a high-spin system.^36,37^ The Fe and Co centers in **2** and **3b** are additionally stabilized by anagostic interactions with one Si-^i^Pr group of the ligand backbone (i.e. in **2**: d_H16–Fe1_ = 2.4508 Å; in **3b**: d_H16–Co1_ = 2.432 Å). This is made feasible by the flexibility of the ligand framework, the central 6-membered [MGeNSiCP] ring forming a boat conformation in those complexes. Notably, such an interaction is absent in Ni complexes **4a** and **4b** (e.g. in **4a**: d_H16–Ni1_ = 2.8036 Å), leading to an essentially planar central 6-membered ring in **4b** whereby no Ni···H interactions are observed below 3 Å (sum of van der Waals radii = 3.60 Å).^38^

**Figure 2:**
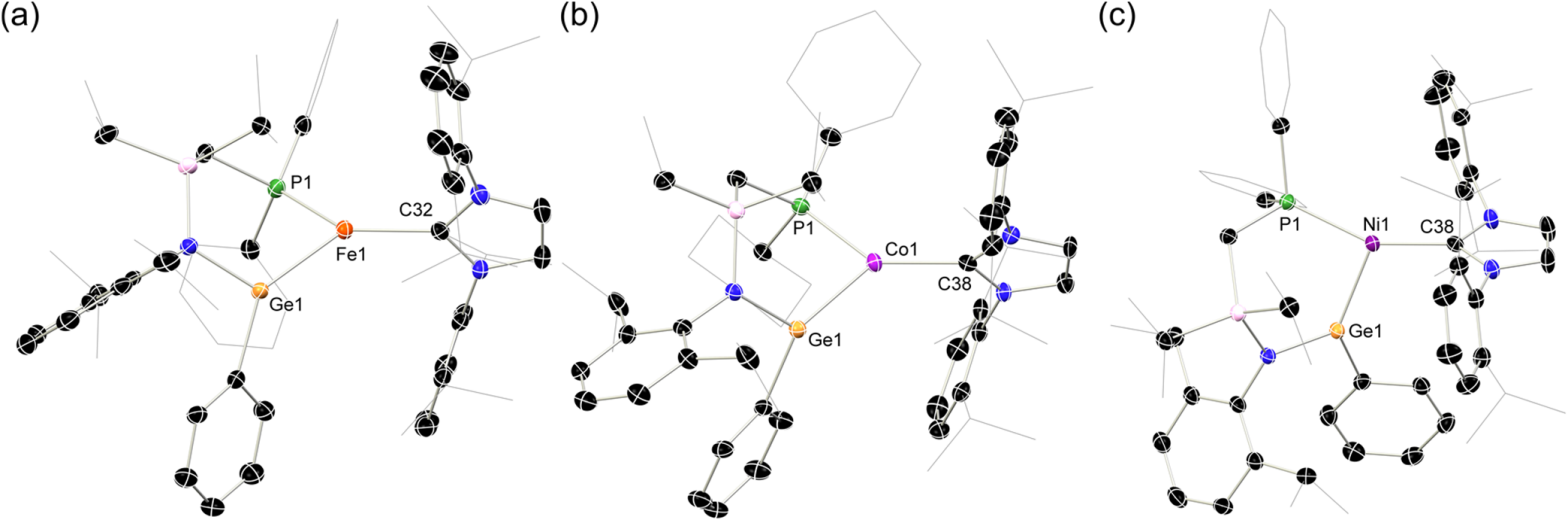
Molecular structure of the cationic part in (a) **2**, (b) **3b**, and (c) **4a**, with ellipsoids at 30% probability, and hydrogen atoms omitted for clarity. Selected bond lengths (Å) and angles (°) for **2**: Ge1–Fe1 2.347(2); Fe1–P1 2.396(4); Ge1–Fe1–P1 89.2(1); P1–Fe1–C_IPr_ 136.3(4); for **3b**: Ge1–Co1 2.326(2); Co1–P1 2.289(3); Ge1–Co1–P1 89.39(9); P1–Co1–C_IPr_ 136.3(3); for **4a**: Ge1–Ni1 2.310(1); Ni1–P1 2.234(2); Ge1–Ni1–P1 91.99(4); P1–Ni1–C_IPr_ 154.3(3).

**Scheme 1:**
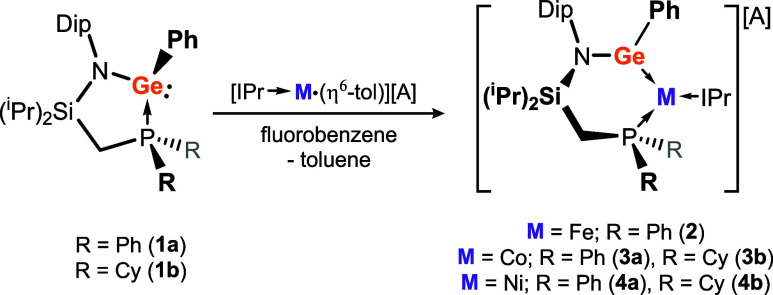
Synthesis of Cationic Germylene M^I^ Complexes **2-4**. A = B­{3,5-(CF_3_)­C_6_H_3_}_4_

The redox nature of compounds **2**, **3**, and **4** was subsequently investigated using cyclic voltammetry (CV) – this led to mixed results, with the majority of reduction and oxidation events being irreversible (Figure 3, and Figures S5, S10, and S15 in Supporting Information). This nevertheless gives insights into the potential stability of reduced (i.e. M^0^) derivatives of Fe and Co complexes **2** and **3**, which we have as yet been unable to access in our laboratories. This contrasts with the Ni derivative, *viz*. ^Ph^L­(Ph)­Ge·Ni·IPr, which is readily generated by the addition of the free germylene to IPr·Ni­(η^6^-tol).^13^ Although a reduction event is observed for cationic Ni system **4a** at 1.772 V, this is ill-defined and irreversible under the given conditions. In contrast, cobalt derivative **3a** shows an essentially reversible reduction event (*E* ^red^ = −1.583 V; *E* ^1/2^ = −1.479 V), which suggests a stable Co^0^ derivative, e.g. ^Ph^L­(Ph)­Ge·Co·IPr. Iron complex **2** lies between these extremes, with a well-defined reduction process observed at −1.752 V, which is irreversible.

**Figure 3:**
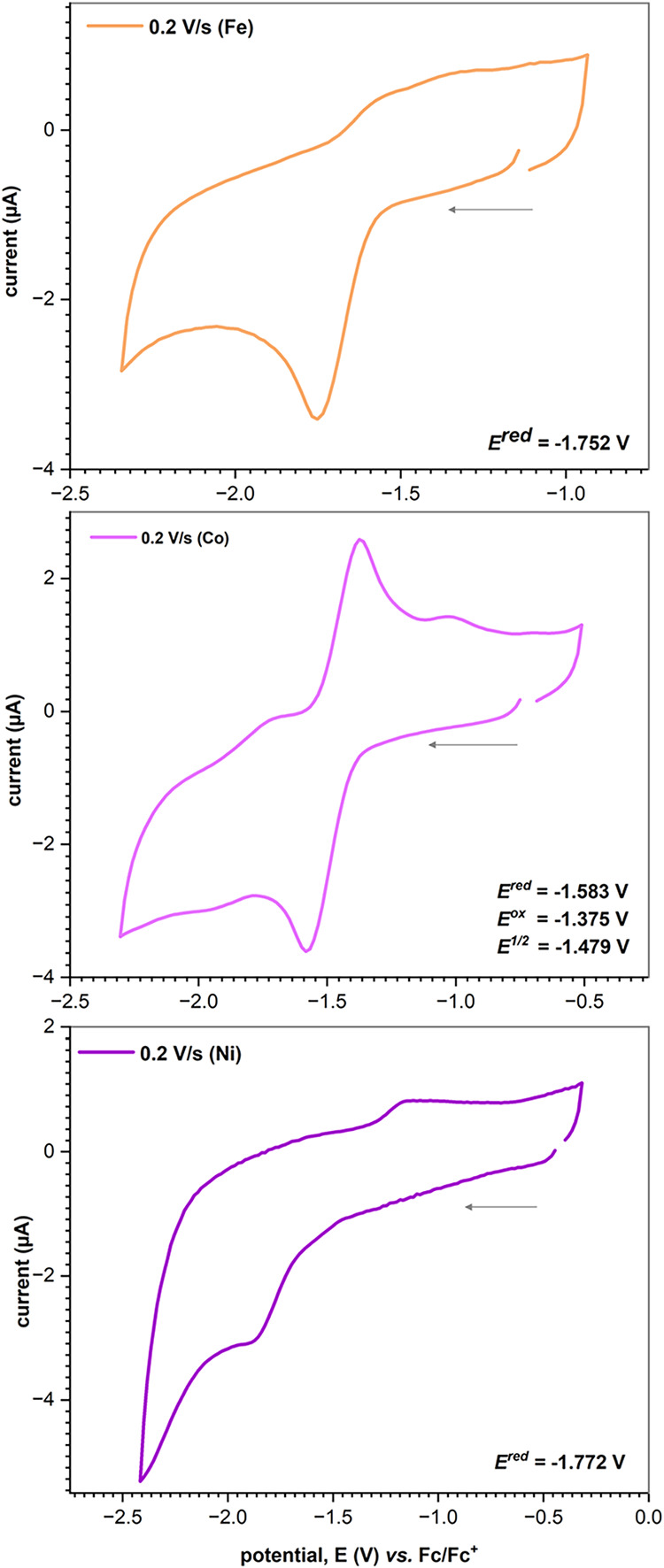
Cyclic voltammograms of complexes **2**, **3a**, and **4a** (from top to bottom), in THF/[N­(*^n^* Bu)_4_]­PF_6_, at a scan rate of 200 mV·s^–1^.

Reactivity studies involving complexes **2–4** yielded poor results. For example, the reaction of Co complex **3a** with NH_3_ led to complex mixtures, from which only a few crystals of the Co^0^ complex [^Ph^L­(NH_2_)­Ge]_2_Co could be isolated, confirming the connectivity in this species (Figure S38). Additionally, all complexes were screened for the catalytic hydrogenation of alkenes, showing generally poor results (see Supporting Information for details), and a propensity to partake in side reactions, *e*.*g*. polymerization. This highlights the challenges of working with open-shell 3d-metal systems.

### Magnetic Properties and Electronic Structure

The homologous series of complexes **2–4** allows for a direct comparison of their electronic nature. As mentioned, all isolated complexes exhibit paramagnetic behavior as evidenced by the ^1^H NMR spectra. From these samples, corresponding effective magnetic moments were determined via the Evans method. Iron­(I) complex **2** can either adopt a low-spin or high-spin d^7^ configuration – a large effective magnetic moment of 4.66 is indicative of an S = ^3^/_2_ ground state, aligning with a high-spin iron­(I) center. SQUID measurements are also in agreement here, yielding an essentially temperature independent magnetic moment of μ_eff_ = 4.8 (Figure S19). significantly higher than the spin-only value of 3.87, and slightly larger than the effective moment of μ_eff_ = 4.19 determined for the toluene-capped starting material, [IPr·Fe­(η^6^-tol)]­[BAr_4_^F^], which we hypothesize may be due to spin–orbit coupling effects of the germylene ligand. Similarly, compounds **3a** and **3b** contain formal d^8^ Co^I^ centers; given their paramagnetic nature they are formally high-spin. This is confirmed with both the Evans method (**3a**: 3.50; **3b**: 3.31) and SQUID magnetometry (**3a**: 3.55), aligning with an S = 1 ground state. This is again slightly higher than the expected spin-only value (2.83), likely due to spin–orbit coupling.^26,22,39^ Turning to the nickel congeners **4a** and **4b**, effective magnetic moments of μ_eff_ = 1.41 and μ_eff_ = 1.53 were found in solution, while a higher value of μ_ef**f**_ = 2.0 was found using SQUID magnetometry. These values are consistent with a d^9^ configuration and an S = 1/2 ground state, the latter being slightly larger than the spin-only value of 1.73.

In comparison, the related toluene-coordinated Ni^I^ complex [IPr·Ni­(η^6^-tol)]­[BAr_4_^F^], exhibits higher moments of μ_eff_ = 2.09 (SQUID) and 2.04 (Evans method), suggesting that **4a** and **4b** undergo a more pronounced quenching of spin–orbit coupling in solution.^32^ Plots of the inverse of the magnetic susceptibility vs. temperature (Figure S19, (c,d)) show *x*-axis intercepts which deviate from 0, leading to Weiss constants (θ_CW_) of −9.6 and −3.8 K for Fe and Ni, respectively. This deviates from the ideal Curie–Weiss behavior observed for their toluene-capped starting materials,^32^ and suggests weak antiferromagnetic coupling effects in **2** and **4**, which may be brought about by M-Ge spin-polarization.

To gain further insights into these observations, the electronic ground states of the novel germylene species described here were investigated using computational methods. In order to gain an accurate depiction of the high-spin ground states, CAS-SCF was used employing truncated models (*viz*. ^Me^L′(Ph)­Ge·M·IXyl; ^Me^L′ = [{Me_2_PCH_2_SiMe_2_}­(Xyl)­N]^−^; IXyl = [(H)­CN­(Xyl)_2_C:]; Xyl = 2,6-Me_2_C_6_H_3_; M = Fe (**2′**), Co (**3′**), Ni (**4′**); see Supporting Information for details) derived from the X-ray crystal structures of the “real” complexes, with heavy-atom positions frozen and hydrogen atom positions refined. DFT starting points used the ωB97x-D3 functional, and the def2-SVP level of theory was used for all calculations, with the chain-of-spheres approximation (RIJCOSX) and the def2/JK auxiliary basis. To gauge the effect of the tetrylene ligand on the ground state of the TM complexes, these calculations were carried out not only on compounds **2–4**, but also on the toluene-capped starting complexes. First, we describe the findings for the cationic moiety in complexes [IPr·M­(η^6^-tol)]­[BAr_4_^F^] (M=Fe, Co, Ni), again using a truncated model (i.e. [IXyl·M­(η^6^-tol)]^+^). Across all calculations described here, energy separations of <0.1 eV are considered as quasi-degenerate.[Fn efn2]

First, all [IXyl·M­(η^6^-tol)]^+^ compounds gave more favorable single-point energies when considered as high-spin complexes, aligning with the experimentally determined magnetic properties. Ground-state wave functions for Co and Ni are represented by a single d^8^ and d^9^ configuration, respectively (Table S2), and are thus considered nondegenerate. The Ni derivative is found to have a low-lying electronic transition 0.197 eV above the zeroth root, also consisting of a single d^9^ configuration. In contrast, we find significant multiconfigurational character for the Fe^I^ ground-state, which is best described as doubly quasi-degenerate:^40^ the zeroth and first roots are separated by just 0.068 eV, with two configurations (≈ 64:36) which we presume arise from the near energetic parity of the d_*xy*_ and d_*x* ^2^ – *y* ^2^_ orbitals (Table S2; *vide infra*). For the three systems, these dominant configurations were combined with NEVPT2-corrected AILFT (*ab initio* ligand field theory)-derived d-orbital energy ordering to construct a qualitative ligand-field picture for chemical interpretation (Figure 4; see Supporting Information for details).[Fn efn1] For Fe^I^ we find a (d_*xy*_,d_*x* ^2^ – *y* ^2^_)_3_,(d_*z* ^2^_)_2_,(d_*xz*_,d_*yz*_)_2_ electronic ground-state, whereby the low-lying degenerate (d_*xy*_,d_*x* ^2^ – *y* ^2^_) subset is triply filled, with a small energy separation of 0.04 eV for this orbital pair. This makes this species a rare example of a stable complex with a non- Aufbau electronic ground state. That is, lower energy orbitals (in this case d_*xy*_ and d_*x* ^2^ – *y* ^2^_ orbitals) remain partially filled while orbitals of higher energy continue to be filled. This rare phenomenon is known to lead to slow magnetic relaxation, and as such is a target in the design of d^7^ species bearing single-molecule magnetic properties.^41^ Notably, this electronic state is very similar to that observed for a previously reported neutral terphenyl-iron­(I) η^6^-benzene complex – this state is key for the magnetic properties of that species, and makes [IPr·Fe­(η^6^-tol)]­[BAr_4_^F^] an interesting candidate for further studies in this regard.^42^

**Figure 4:**
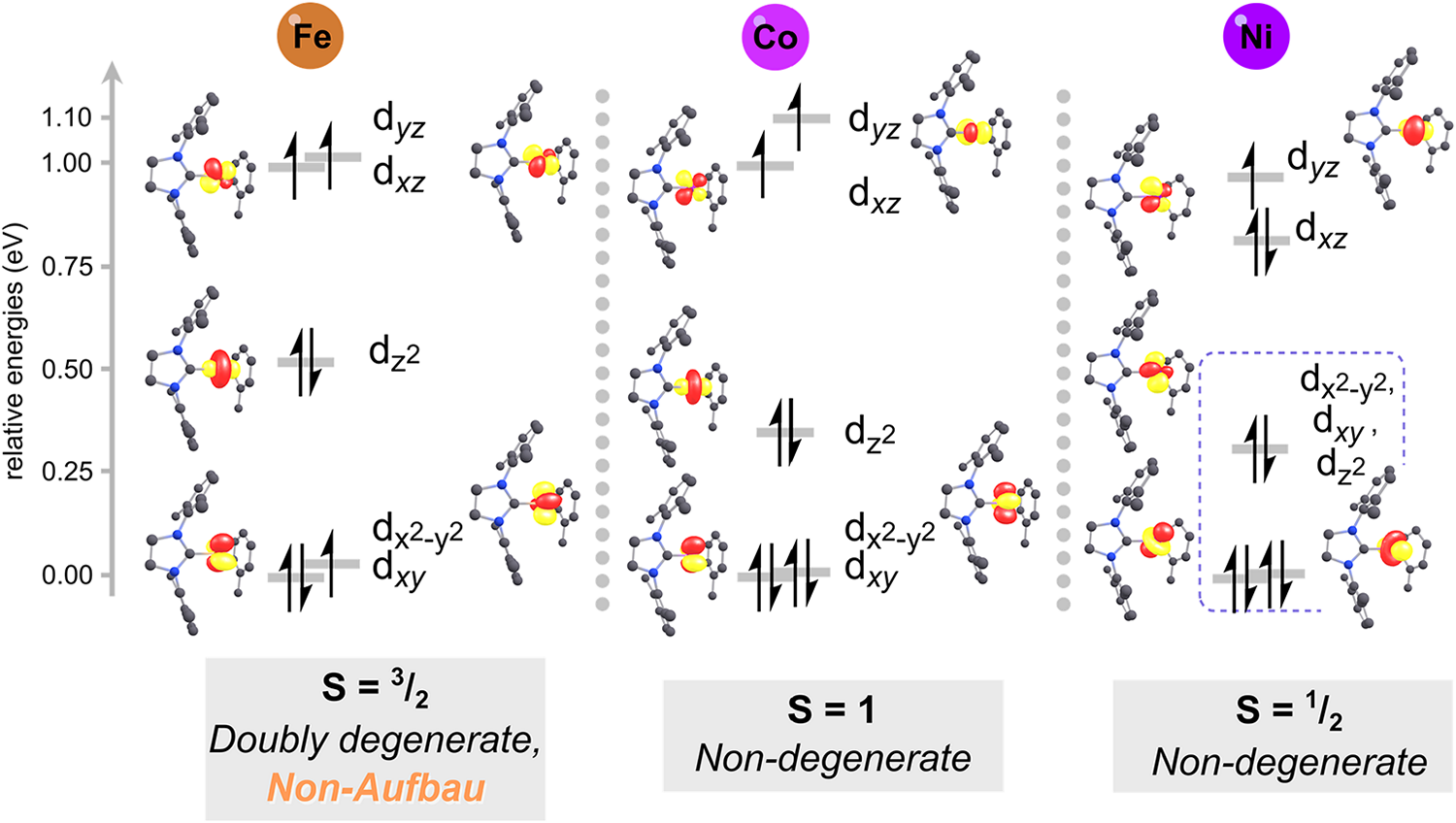
CAS-SCF/NEVPT2 AILFT-derived orbital occupancies, using truncated model complexes [IXyl·M­(η^6^-tol)]^+^, for M = Fe, Co, and Ni. The active space in each case used the metal d-orbitals and the respective d-electron count (i.e. Fe: (7,5); Co: (8,5); Ni: (9,5)). Relative orbital positioning is qualitatively based upon AILFT energies (absolute energies are tabulated in the Supporting Information).

For the corresponding Ni complex, orbital mixing is observed for the d_*z* ^2^_, d_*xy*_, and d_*x* ^2^ – *y* ^2^_ orbitals, all of which are doubly filled, and the aforementioned low-lying electronic transition is represented by a d_*xy*_→d_*yz*_ transition of 0.186 eV. The Co^I^ cation demonstrates a nondegenerate ground state, with a (d_*xy*_,d_*x* ^2^ – *y* ^2^_)_4_,(d_*z* ^2^_)_2_,(d_*xz*_,d_y*z*_)_2_ orbital occupancy. Comparing orbital energies for all three metals, an increased separation of orbitals aligned with the *z*-axis is observed with an increasing electron count (*e*.*g*. ΔE­(d_*xz*_d_*yz*_) for Fe: 0.021 eV; for Co: 0.154 eV; for Ni: 0.186 eV). This aligns with the distortion/slipping of the toluene binding in this complex, the degree of which again increases across the series Fe < Co < Ni, which we presume is due to repulsive interactions between these filled orbitals and the arene ligand.

As described above, computational studies pertaining to complexes **2–4** utilized truncated model species **2**′, **3**′, and **4**′ (see Supporting Information for details). Here, AILFT calculations were not included as this would negate the inclusion of the Ge centered p-orbital. Thus, relative orbital levels are given by occupancy (Figure 5). Again, high-spin (i.e. quartet (Fe) and triplet (Co)) ground states were found to be the most stable. All calculations concluded with 6 active orbitals, being the 5 d-orbitals and the vacant p-orbital at Ge, giving the active spaces of (7,6) [Fe], (8,6) [Co], and (9,6) [Ni] (see Supporting information for details). These CAS-SCF calculations aimed to define any spin-delocalization/polarization on Ge, and thus give insights into the direct electronic effect of the tetrylene′s electron deficiency within these complexes. The stand-out system here is again Fe complex **2′**: rather than a break in degeneracy upon tetrylene coordination, a doubly quasi-degenerate ground state is maintained with the first root just 0.018 eV above the ground state, in addition to a now very low energy second root lying only 0.108 eV (2.49 kcal·mol^–1^) above the ground state (Table 1). This electronic situation seems to be due to the similar energy of the d_*z* ^2^_, d_*xz*_, and d_*yz*_ orbitals, borne out by the multiconfigurational nature of the first two roots, each dominated by a distinct pair of configurations (i.e. zeroth root: 35% 211210, 31% 212110; first root: 41% 221110, 23% 212110). Significant spin-polarization is found from the doubly occupied d_*x* ^2^ – *y* ^2^_ orbital to the vacant p-orbital on Ge, with a formal occupancy of the latter calculated at 0.44e, and spin-polarization at Ge of −0.29 (spin at Fe = 3.31). This justifies the negative experimentally determined Weiss constant for **2** (θ_CW_ = −9.6 K), i.e. being representative of antiferromagnetic coupling between Fe and Ge. This spin-polarization (or anti- ferromagnetic coupling) value potentially contributes to the large magnetic moment observed for **2** of μ_eff_ = 4.8. This at first seems counterintuitive, i.e. that antiferromagnetic coupling would *increase* μ_eff_. However, this effect is exclusive from the iron-centered unpaired electrons, and rather arises from a doubly filled orbital, and therefore does not quench the magnetism arising from the former unpaired electrons. We thus describe the spin-polarization as additive. While this does not strictly pertain to spin–orbit coupling, ligand-centered spin is known to increase magnetic anisotropy and spin–orbit coupling, which in turn increases μ_eff_.^43^ A lower degree of spin-polarization is observed in Ni complex **4′** ([Ni: 1.16; Ge: −0.15]; p-orbital population of 0.43e), which we presume is due to the lower spin quantum number of this complex, and again justifies the smaller observed Weiss constant for this system (θ_CW_ = −3.8 K). As in above-described Ni^I^ toluene complex, significant mixing of the d-orbitals in **4′** is observed. Still, it is interesting to note that this species bears two degenerate d-orbitals, despite being T-shaped, whereby a typical ligand field for this geometry would favor 5 nondegenerate d-character orbitals.^34,35^ This is presumably due to the back-bonding interaction with the Ge^II^ center (or the above-described antiferromagnetic coupling), and highlights an important electronic effect of this ligand class. Similarly, and despite its even (i.e. d^8^) electron count, Co^I^ complex **3′** exhibits a low-lying excited state, with an energy separation of the first two roots being 0.21 eV (4.8 kcal·mol^–1^; Table 1). These insights demonstrate the utility of tetrylenes in tuning the magnetic anisotropy of an open-shell TM species; further work is required here to better define such effects, which we aim to explore moving forward.

**Figure 5:**
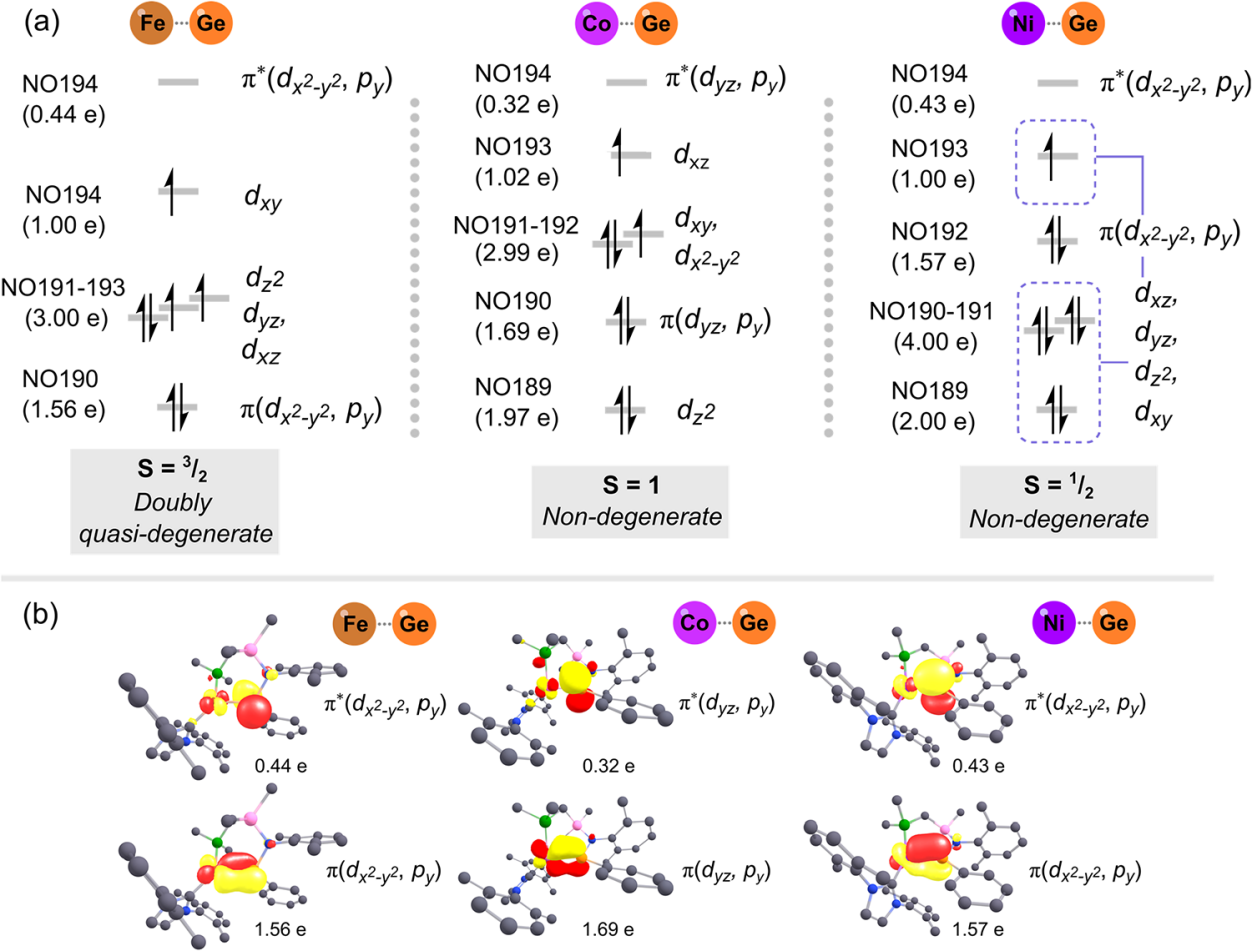
(a) CAS-SCF/NEVPT2 derived natural orbital (NO) occupancies for the cationic species **2′-4′**, and (b) graphical representations of the NOs involved in spin-polarization (remaining active space NOs are visualized in the Supporting Information). NOs are ordered by occupation number, and not by absolute energy; this is therefore qualitative.

**Table 1:** Summary of the CAS-SCF/NEVPT2 Ground-State Calculations for **2′-4′**

	relative energy (eV)			state occupancies^a^					
root	Fe	Co	Ni	Fe		Co		Ni	
**0**	0.000	0.000	0.000	0.35181	211210	0.58291	222110	0.73154	222210
				0.31011	212110	0.19183	221210	0.1665	222012
				0.08016	111211	0.08082	202112	0.09622	222111
				0.07144	112111	-	-	-	-
**1**	0.018	0.215	-	0.41263	221110	0.57741	221210	-	-
				0.22656	212110	0.19788	222110	-	-
				0.0919	121111	0.07833	201212	-	-
				0.07844	21112	-	-	-	-
**2**	0.108	-	-	0.28054	211210	-	-	-	-
				0.25494	221110	-	-	-	-
				0.15551	212110	-	-	-	-

a Only states with an occupancy above 7% are listed here. All states are given in the Supporting Information.

## Conclusion

Herein, we have described a rational synthesis of open-shell 3d-metal germylene complexes with intriguing electronic properties. All species adopt high-spin configurations with one to three unpaired electrons, progressing from nickel to iron. Their magnetic moments, determined via the Evans method and SQUID magnetometry, revealed exceptionally high effective values for the iron- and cobalt-containing complexes **2a** and **3b**, respectively. A deeper dive into their electronic structure using CAS-SCF calculations indicates a remarkable doubly quasi-degenerate ground state for Fe, moving to a nondegenerate Ni system, with notably low-lying excited states for both Fe and Co. In all cases a significant degree of spin-polarization on the germylene ligand is found, which we correlate with a degree of spin–orbit coupling in these systems, ultimately leading to larger than expected values for μ_eff_. While poor catalytic performance is found, perhaps due to the highly reactive nature of these open-shell species, these findings give key insights into the effect of tetrylene coordination on the magnetic properties of high-spin TM species. We hope to further expand on these observations in due course.

## Experimental Section

### General Considerations

All experiments and manipulations were carried out under a dry oxygen-free argon atmosphere using standard Schlenk techniques or in an MBraun inert atmosphere glovebox containing an atmosphere of high-purity argon. THF and diethyl ether were dried by distillation over a sodium/benzophenone mixture and stored over activated 4Å molecular sieves. C_6_D_6_ was dried, degassed and stored over a potassium mirror. All other solvents were dried over activated 4Å molecular sieves and degassed prior to use. ^Ph^LGePh,^44^ ^Cy^LGePh,^45^ and [IPr·M­(η^6^-tol)]­[BAr_4_^F^] (M = Fe, Co, Ni)^32^ were synthesized according to known literature procedures. All other reagents were used as received. NMR spectra were recorded on a Bruker AV 400 Spectrometer. The ^1^H and ^13^C­{^1^H} NMR spectra were referenced to the residual solvent signals as internal standards. Liquid Injection Field Desorption Ionization Mass Spectrometry (LIFDI-MS) was measured directly from an inert atmosphere glovebox with a Thermo Fisher Scientific Exactive Plus Orbitrap equipped with an ion source from Linden CMS.^46^ Absorption spectra (UV/vis) were recorded on an Agilent Cary 60 UV/vis spectrophotometer. Elemental analyses (C, H, N) were performed with a combustion analyzer (elementar vario EL, Bruker). Cyclic Voltammetry was carried out with a PalmSens4 potentiostat with PSTrace Software, using 3 mm diameter glassy carbon disk electrodes (PalmSens, Houten Netherlands) as a working electrode, and Pt wire as the counter electrode. Prior to use, electrodes were polished with 0.05 μm alumina suspensions. Ag/AgSbF_6_ (0.01 M if PhF) was used as the reference electrode, using the construction reported by Halter and co-workers.^47^ [^n^Bu_4_N]­[PF_6_] was used as the electrolyte (0.1 M). CV measurements were performed in an inert atmosphere glovebox, under an Ar atmosphere. Potentials are reported with reference to an internal standard of ferrocenium/ferrocene. SQUID magnetic susceptibility measurements were carried out with a Quantum-Design MPMS3 SQUID magnetometer in VSM mode and in an applied magnetic field of 10,000 Oe at zero-field cooled (ZFC) and field cooled (FC). A brass sample holder was used for all measurements and the magnetic susceptibility data was collected in the temperature range between 2 and 300 K. Samples were prepared under an inert atmosphere using VSM powder capsules, previously dried at 110 °C for several days. For a background correction, the magnetic moment of a capsule containing no sample was subtracted from the magnetic moment obtained for the sample, measured under the same conditions. The data were corrected for diamagnetic contributions using tabulated Pascal constants^48^ and plotted as μ_eff_ and 1/χ_p_ versus *T* where, χ_p_ represents χ_MOL_(sample) – χ_DIA_(Pascal correction).

### General Procedure for the Synthesis of Germylene Complexes 2–4

To a flask containing [IPr·M­(η^6^-tol)]­[BAr_4_^F^] (1.0 equiv) was added fluorobenzene (0.1 mL·mg^–1^), and the resulting solution cooled to −30 °C with stirring. Subsequently, a solution of ^R^L­(Ph)­Ge (1.0 equiv) in fluorobenzene (0.1 mL·mg^–1^) was added dropwise over the course of 5 min. After stirring for 10 min at this temperature, the reaction mixture was removed from the cold bath, and allowed to warm to room temperature with stirring, leading to intensely colored solutions in all cases. All volatiles were then removed *in vacuo*, and the residue triturated with pentane (2 × 50 mL·g^–1^), leading to colorful powders which were dried *in vacuo*, and identified as the analytically pure target compounds.

### [^Ph^L­(Ph)­Ge·Fe·IPr]­[BAr_4_^F^], **2a**.

This compound was obtained via the general procedure using ^Ph^L­(Ph)Ge (350 mg, 0.55 mmol, 1.0 equiv) and [IPr·Fe­(η^6^-tol)]­[BAr_4_^F^] (768 mg, 0.55 mmol, 1.0 equiv), and was isolated as a purple solid (823 mg, 0.42 mmol, 77%). Crystals suitable for X-ray analyses were obtained by recrystallization from a concentrated fluorobenzene solution, layered with pentane at 7 °C after 2 days.

^**1**^ **H NMR** (C_6_D_6_/PhF (3:1), 400 MHz, 298 K): −19.80, −15.97, −13.91, −10.49, −9.02, −3.92, −2.39, −1.32, −0.91, 0.28, 0.87, 1.44, 1.80, 2.93, 3.11, 4.19, 5.71, 7.62, 7.71, 8.34, 9.83, 13.72, 14.96, 15.80, 16.74, 20.36, 30.37.

**Magnetic moment** (C_6_D_6_/PhF (3:1), 400 MHz, 298 K): 4.66 μ_B_; (SQUID; crystalline solid, 298 K): 4.8 μ_B_.

**MS/LIFDI-HRMS** found (calcd.) *m*/*z*: 1083.4749 (1083.4378) for [M-BAr_4_^F^]^+^.

**λ**_**max**_, nm (*ε*, Lcm^–1^ mol^–1^): 547 (790), 372 (1508).

**Anal. Calcd**. for C_96_H_96_BF_24_FeGeN_3_PSi: C, 59.25%; H, 4.97%; N, 2.16%; found C, 57.19%; H, 4.78%; N, 2.24%.

### [^Ph^L­(Ph)­Ge·Co·IPr]­[BAr_4_^F^], **3b**

This compound was obtained via the general procedure using ^Cy^L­(Ph)Ge (300 mg, 0.46 mmol, 1.0 equiv) and [IPr·Co­(η^6^-tol)]­[BAr_4_^F^] (648 mg, 0.46 mmol, 1.0 equiv), and was isolated as a dark pink solid (678 mg, 0.35 mmol, 75%). Crystals suitable for X-ray analysis were obtained by recrystallization from a concentrated fluorobenzene solution layered with pentane at room temperature, after 1 day.

^**1**^ **H NMR** (C_6_D_6_/PhF (3:1), 400 MHz, 298 K): −29.55, −27.74, −21.02, −12.36, −10.11, −7.01, −5.58, −4.60, −3.71, −3.13, −2.97, −2.62, −1.77, −1.26, −1.16, −1.15, −0–93, −0.10, 0.28, 0.31, 0.52, 0.80, 1.54, 1.83, 2.85, 3.88, 4.53, 4.67, 4.91, 5.16, 5.73, 6.10, 6.25, 7.63, 8.26, 8.33, 8.34, 10.02, 10.91, 13.41, 16.54, 18.58, 21.31, 23.33, 24.02, 26.16.

**Magnetic moment** (C_6_D_6_/PhF (3:1), 400 MHz, 298 K): 3.31 μ_B_.

**MS/LIFDI-HRMS** found (calcd.) *m*/*z*: 1098.5729 (1098.5297) for [M-BAr_4_^F^]^+^.

**λ**_**max**_, nm (*ε*, Lcm^–1^ mol^–1^): 625 (438), 548 (628), 364 (1740).

**Anal. Calcd**. for C_96_H_108_BCoF_24_GeN_3_PSi: C, 58.79%; H, 5.55%; N, 2.14%; found C, 59.75%; H, 5.84%; N, 2.34%.

### [^Ph^L­(Ph)­Ge·Ni·IPr]­[BAr_4_^F^], **4a**

This compound was obtained via the general procedure using ^Ph^L­(Ph)Ge (500 mg, 0.78 mmol, 1.0 equiv) and [IPr·Ni­(η^6^-tol)]­[BAr_4_^F^] (1.1 g, 0.78 mmol, 1.0 equiv), and was isolated as a dark red solid (1.27 g, 0.65 mmol, 83%). Crystals suitable for X-ray analyses were obtained by recrystallization from a concentrated fluorobenzene solution, layered with pentane at room temperature.

^**1**^ **H NMR** (C_6_D_6_/PhF (3:1), 400 MHz, 298 K): −5.36, 0.28, 1.44, 1.85, 3.20, 7.67, 7.83, 8.41, 9.52, 18.07, 24.08.

**Magnetic moment** (C_6_D_6_/PhF (3:1), 400 MHz, 298 K): 1.41 μ_B_; (SQUID; crystalline solid, 298 K): 2.0 μ_B_.

**MS/LIFDI-HRMS** found (calcd.) *m*/*z*: 1085.4733 (1085.4371) for [M-BAr_4_^F^]^+^.

**λ**_**max**_, nm (*ε*, Lcm^–1^ mol^–1^): 390 (1556).

**Anal. Calcd**. for C_96_H_96_BF_24_GeN_3_NiPSi: C, 59.16%; H, 4.97%; N, 2.16%; found C, 57.16%; H, 4.94%; N, 2.04%.

### [^Cy^L­(Ph)­Ge·Ni·IPr]­[BAr_4_^F^], **4b**

This compound was obtained via the general procedure using ^Cy^L­(Ph)Ge (400 mg, 0.62 mmol, 1.0 equiv) and [IPr·Ni­(η^6^-tol)]­[BAr_4_^F^] (864 mg, 0.62 mmol, 1.0 equiv), and was isolated as a red solid (954 mg, 0.49 mmol, 79%). Crystals suitable for X-ray analysis were obtained by recrystallization from a concentrated fluorobenzene solution, layered with pentane stored at room temperature for 3 days.

^**1**^ **H NMR** (C_6_D_6_/PhF (3:1), 400 MHz, 298 K): −38.82, −32.50, −29.51, 22.48, −13.01, −12.53, −9.09, −7.98, −7.20, −6.49, −4.52, −3.65, −3.13, −2.11, −0.20, −0.09, 0.13, 1.44, 1.64, 2.87, 3.24, 3.44, 3.68, 4.01, 4.19, 4.44, 4.62, 5.52, 5.87, 6.05, 6.30, 7.62, 8.06, 8.11, 8.32, 9.18, 10.21, 10.66, 11.23, 11.27, 12.53.

**Magnetic moment** (C_6_D_6_/PhF (3:1), 400 MHz, 298 K): 1.53 μ_B_.

**MS/LIFDI-HRMS** found (calcd.) *m*/*z*: 1097.5664 (1097.5310) for [M-BAr_4_^F^]^+^.

**λ**_**max**_, nm (*ε*, Lcm^–1^ mol^–1^): 344 (5064).

**Anal. Calcd**. for C_96_H_108_BF_24_GeN_3_NiPSi: C, 58.80%; H, 5.55%; N, 2.14%; found C, 58.24%; H, 5.62%; N, 2.09%.

### X-ray Crystallographic Details

Single crystals of **2**, **3a**, **4a**, and **4b** suitable for X-ray structural analysis were mounted in perfluoroalkyl ether oil on a nylon loop and positioned in a 150 K cold N_2_ gas stream. Data collection was performed with a STOE StadiVari diffractometer (Mo Kα radiation) equipped with a DECTRIS PILATUS 300 K detector. Structures were solved by Direct Methods (SHELXS-97),^49^ or using SHELXT-16,^50^ and refined by full-matrix least-squares calculations against F^2^ (SHELXL-2018).^51^ The positions of the hydrogen atoms were calculated and refined using a riding model. All non-hydrogen atoms were treated with anisotropic displacement parameters. Crystal data, details of data collections, and refinements for all structures can be found in their CIF files, which are available free of charge via www.ccdc.cam.ac.uk/data_request/cif, and are summarized in Table S1.

### Computational Methods and Details

Computational experiments were performed using the ORCA 5.0.4 program, on the cationic parts of complexes described in the main text.^52^ This utilized truncated models in which ^i^Pr and P-*Ph* groups are replaced with methyl substituents (see Figure S36 in Supporting Information), due to the size of the real structures leading to nonfeasible computation times for CAS-SCF calculations. Initial DFT optimization of hydrogen positions, with heavy-atom position locked, was carried out at the ωB97XD level with the def2-SVP basis set for all atoms.^53–56^ The RIJCOSX approximation was also used throughout.^57^ Molecular coordinates are included as a separate file. CAS-SCF calculations were carried out using DFT orbitals as an initial guess. This involved first fragmenting the complexes into the respective metal atom, and remaining ligand fragments, and recombining output.gbw files using the ORCA ‘MergeFrag′ program. This merged.gbw is used as the initial guess in each case. For all [IXyl·M­(η^6^-tol)]^+^ complexes the active space comprises the five d-orbitals; for **2′-4′**, the vacant Ge-centered p-orbital is included, with the following work-flow: the initial CAS-SCF calculations for complexes **2′-4′** utilized active spaces consisting of the d-orbitals (i.e. 7,5, 8,5 and 9,5). The active space was expanded through a search for partner orbitals (PMOs), using the “*intorb pmos*” and “*extorb pmos*” keywords. In all cases, this identified only the Ge-centered vacant p-orbital as a partner, which was subsequently included in the active space. Interactions with other ligand orbitals were not found. Given the high-spin nature or otherwise (partially) filled d-orbitals in all systems, the lone electron pair at Ge is presumed to coordinate the *d*-block metal′s vacant s-orbital. These two orbitals are therefore not included in the active space in these complexes. Thus, for [IXyl·M­(η^6^-tol)]^+^ species active spaces of (7,5) (Fe), (8,5) (Co), and (9,5) (Ni) are employed, and (7,6), (8,6), and (9,6) for complexes **2′-4′**, respectively. For these latter complexes, the sixth orbital is the vacant p-orbital at Ge. Where ground-state near degeneracy/low-lying excited states were observed (i.e. roots within ∼0.25 eV of the zeroth root), the additional roots were included in the final ground-state calculation. In all cases, the final calculation included n-electron valence state perturbation theory (NEVPT2) corrections, yielding the final reported results. A summary of contributions to the wavefuntions for all 6 calculated species if given in Tables S2–S5 of the Supporting Information.

## Supplementary Material





## Footnotes

1 The term “quasi-degenerate” is used, and not “degenerate”, as the latter strictly refers to orbitals/states of the same energy. Given standard error margins in CASSCF-NEVPT2 calculations, calculated energies of <0.1 eV would suggest significant population of states within the accessible energetic window at ambient temperature.

2 Positive comparison of the CAS-SCF and AILFT orbital outputs would suggest that this extrapolation is feasible (seeFigure S40in Supporting Information).

## Notes

Coordinates_LGePh_Mcats.xyz. (TXT)
Printed spectra for all new compounds; details regarding X-ray crystallography; and further computational details (PDF)
