# Supplementary material for: On the Electronic Nature of Open-Shell Germylene Complexes of MI Cations (M = Fe, Co, Ni)
Source: Inorg Chem. 2026 Jun 25;65(27):15326–34. doi: 10.1021/acs.inorgchem.6c01906 (PMC13370851; doi:10.1021/acs.inorgchem.6c01906)
Supplement: Supplementary file 2 [file ic6c01906_si_002.pdf]

Supporting Information

On the Electronic Nature of Open-Shell Germylene Complexes of M^{I} Cations (M = Fe, Co, Ni)

Annika Schulz,[a] Jonas L. Gilch,[a] Konstantin B. Krause,[b] Christian Limberg,[b] and Terrance J. Hadlington*,[a]

[a] Fakultät für Chemie, School of Natural Sciences, TU München, Lichtenberg Strasse 4, 85749 Garching, Germany

Email: terrance.hadlington@tum.de

[b] Institut für Chemie, Humboldt-Universität zu Berlin, Brook-Taylor-Strasse 2, 12489 Berlin, Germany

1. Experimental Procedures and data S2

Printed spectra.. S2

Alkene hydrogenation catalysed by complexes **2-4**.. S11

2. X-ray crystallographic details.. S22

3. Computational methods and details.. S25

4. References.. S30

1. Experimental methods and data

Printed graphical data

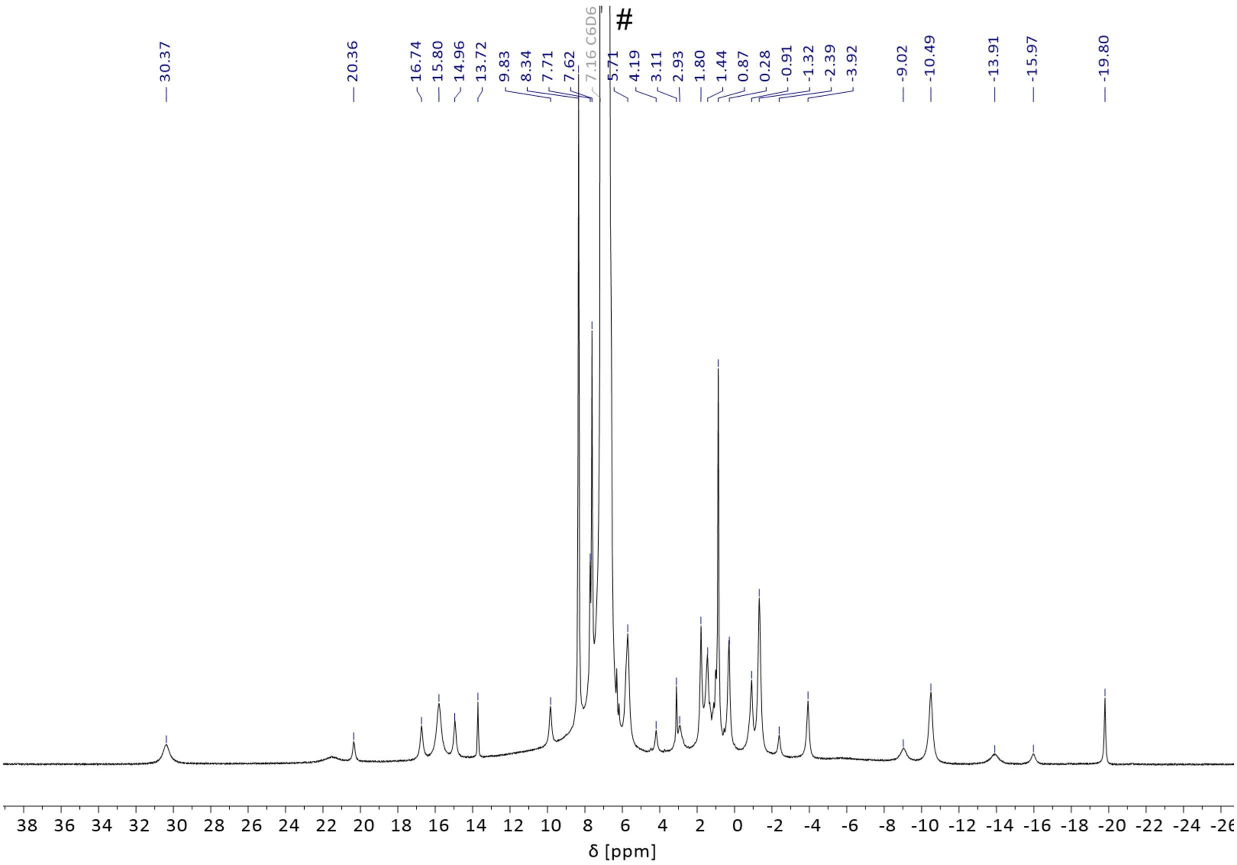

Figure S1. ^{1}H NMR spectrum of **2** in a C_6D_6/PhF (3:1) solution. # marks PhF.

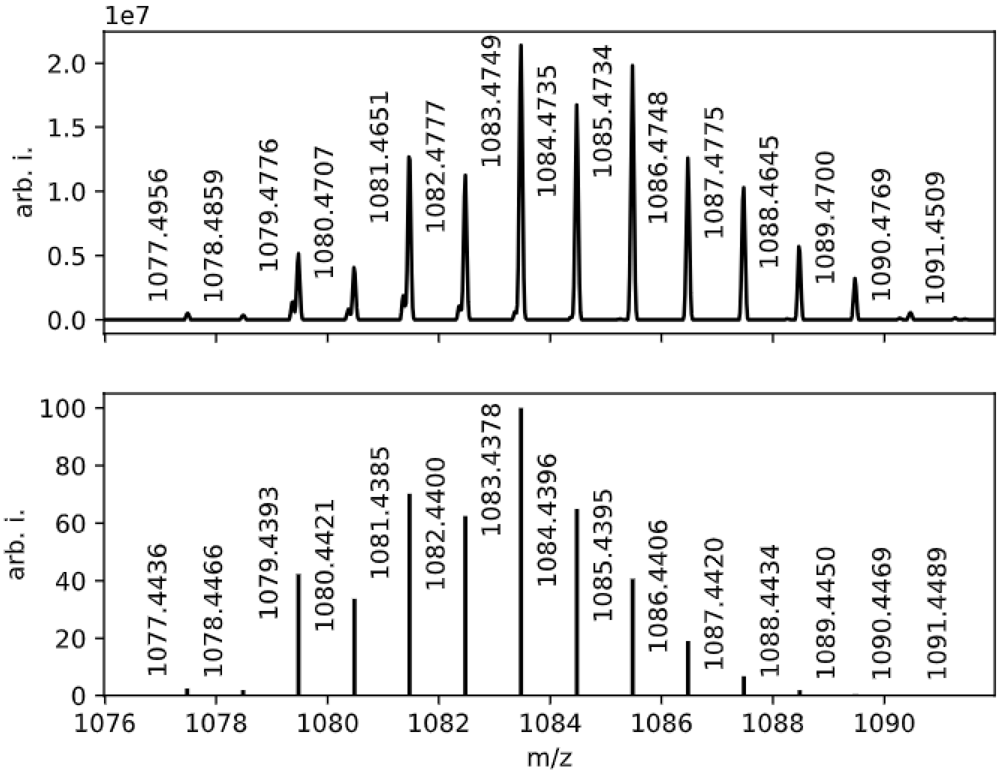

Figure S2. Top: Cutout from LIFDI/MS of **2;** Bottom: Calculated MS spectrum of $[M\text{-}BAr_4^F]^+$.

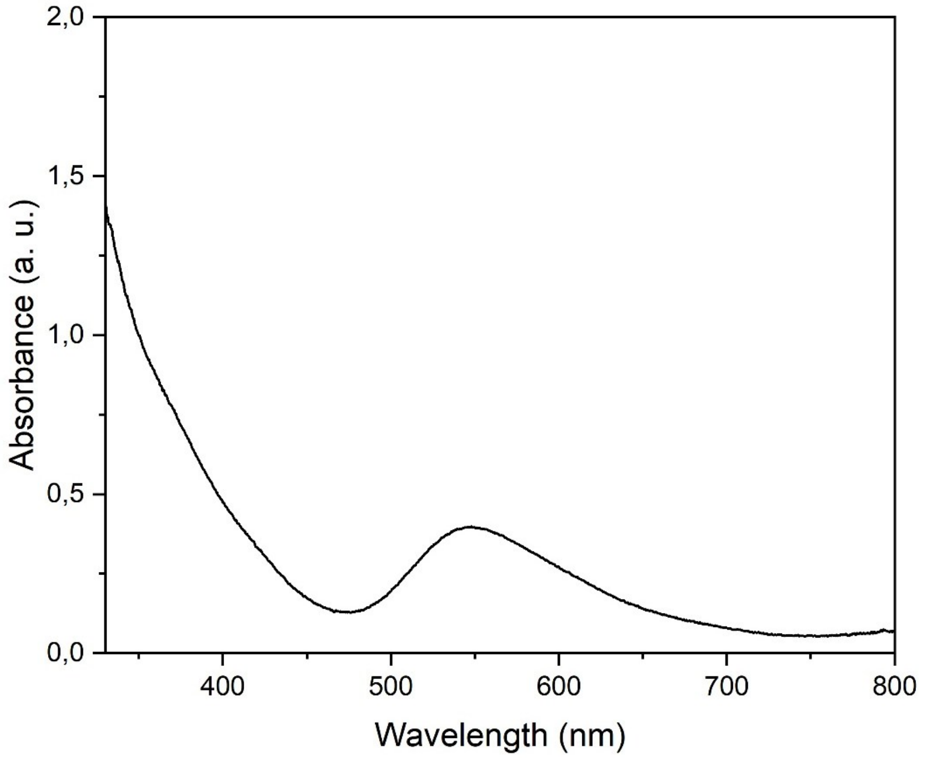

Figure S3. UV/Vis spectrum of a 5x10^{-4} M solution of **2** in toluene/PhF (3/1) at ambient temperature.

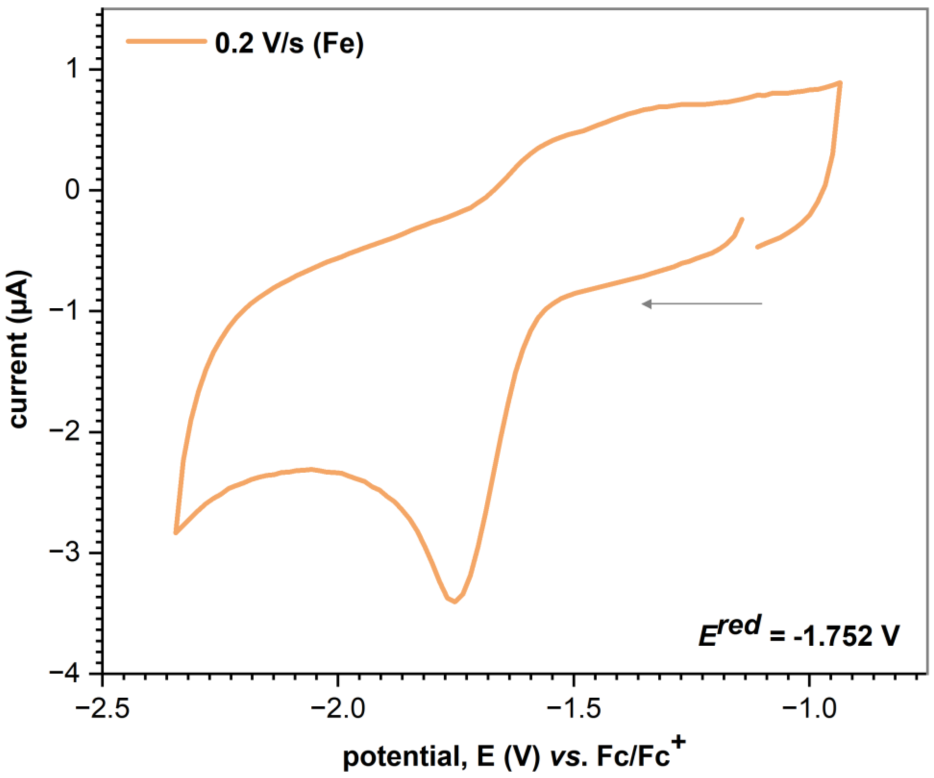

Figure S4. Cyclic voltammogram of a 3 mM solution of **2** in THF under Ar (*i.e.* in a glove box); 0.1 M [^{n}Bu$_4$N][PF$_6$]; 0.2 V·s^{-1}.

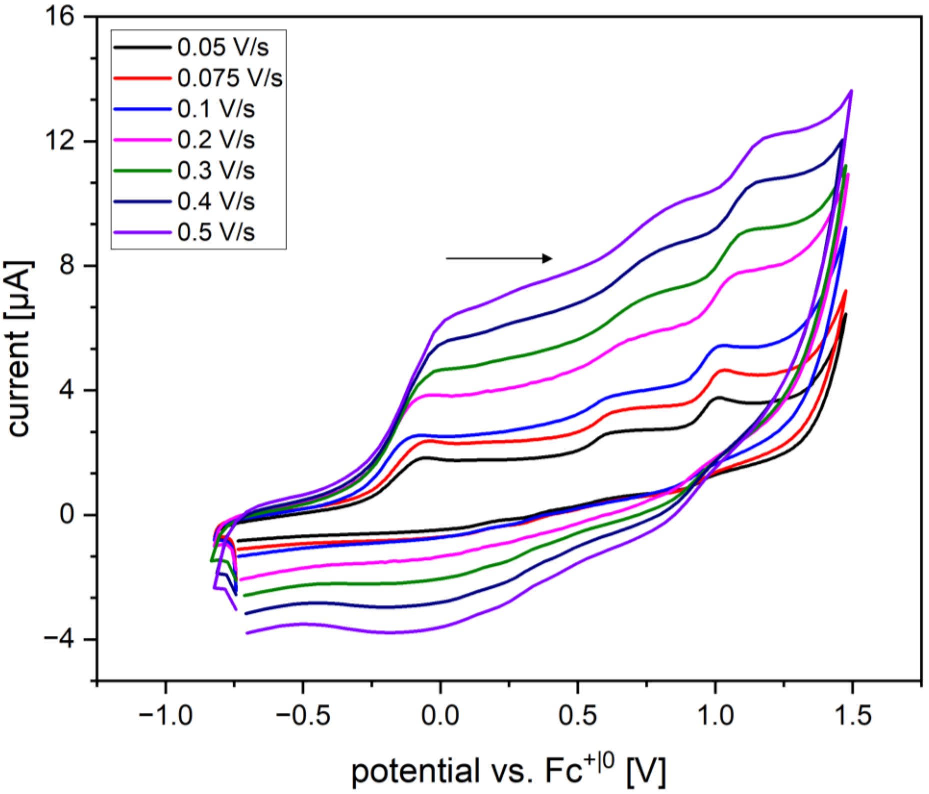

Figure S5. Stack-plot of oxidative cyclic voltammograms of a 3 mM solution of **4a** in THF under Ar (*i.e.* in a glove box); 0.1 M [^{n}Bu$_4$N][PF$_6$], wit varying scan rate.

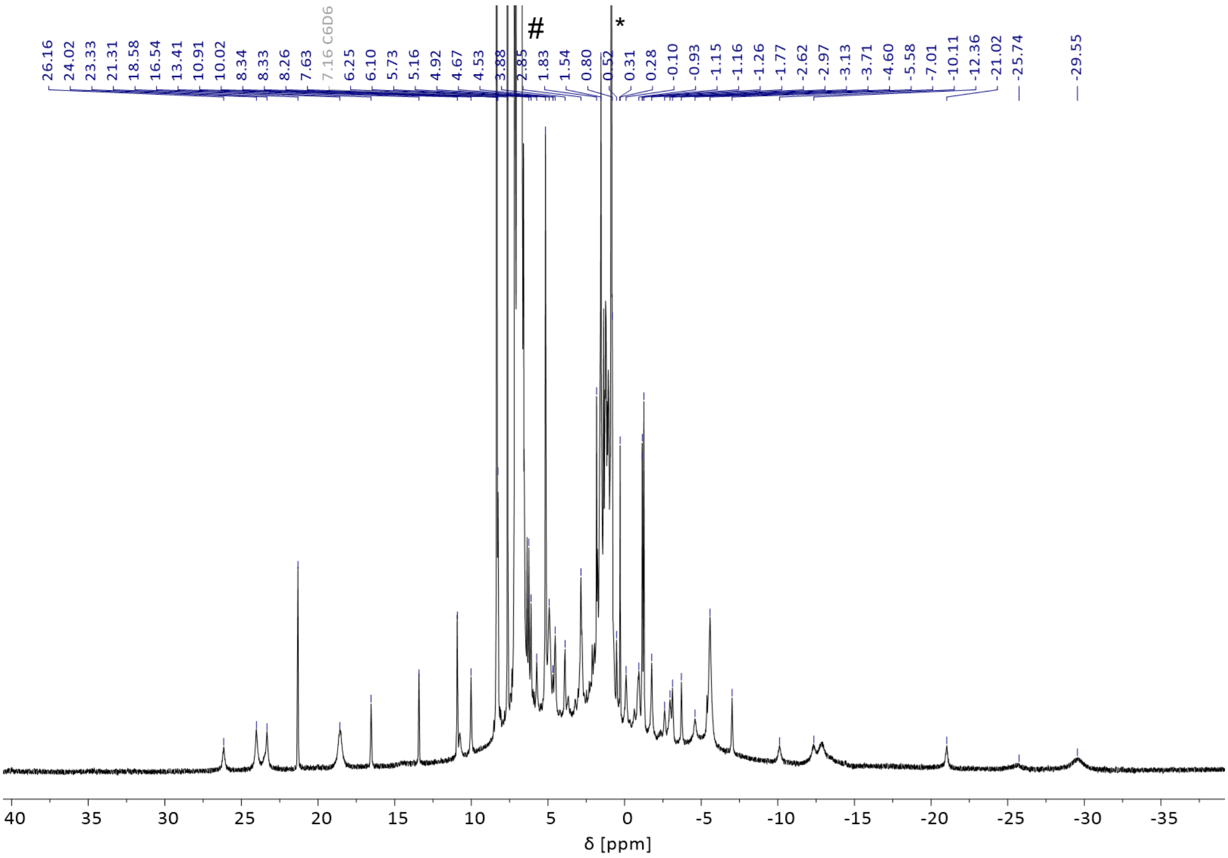

Figure S6. ^{1}H NMR spectrum of **3b** in a C_6D_6/PhF (3:1) solution. # marks PhF, while * marks small amounts of residual pentane.

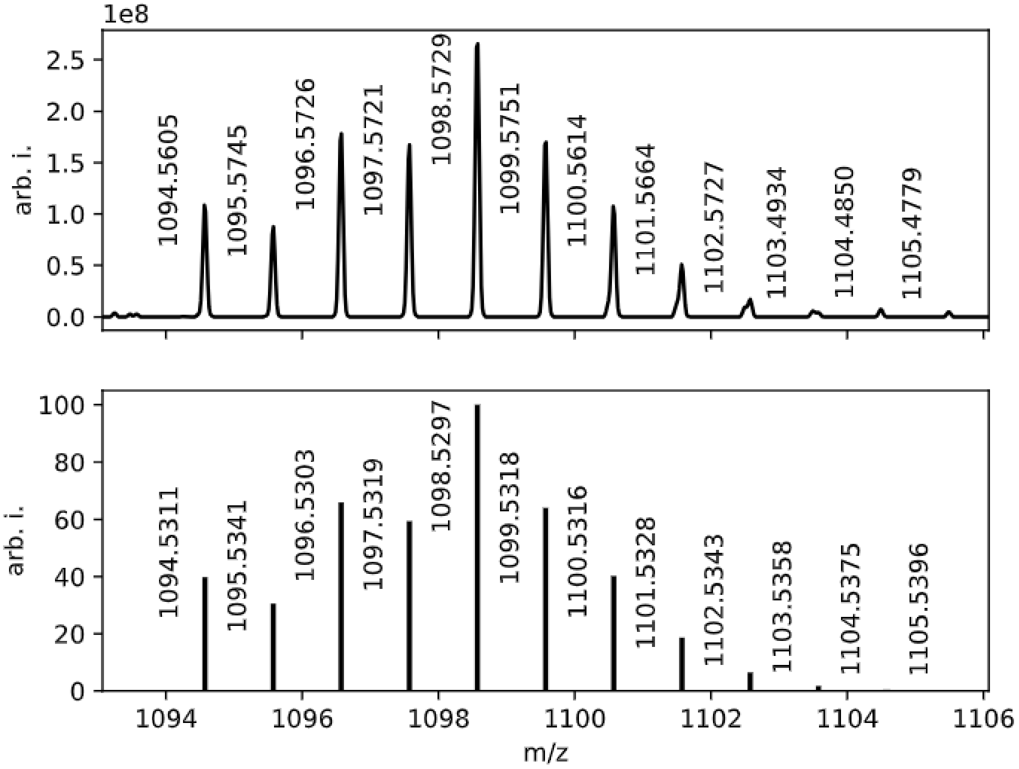

Figure S7. Top: Cutout from LIFDI/MS of **3b**; Bottom: Calculated MS spectrum of $[M\text{-}BAr_4^F]^+$.

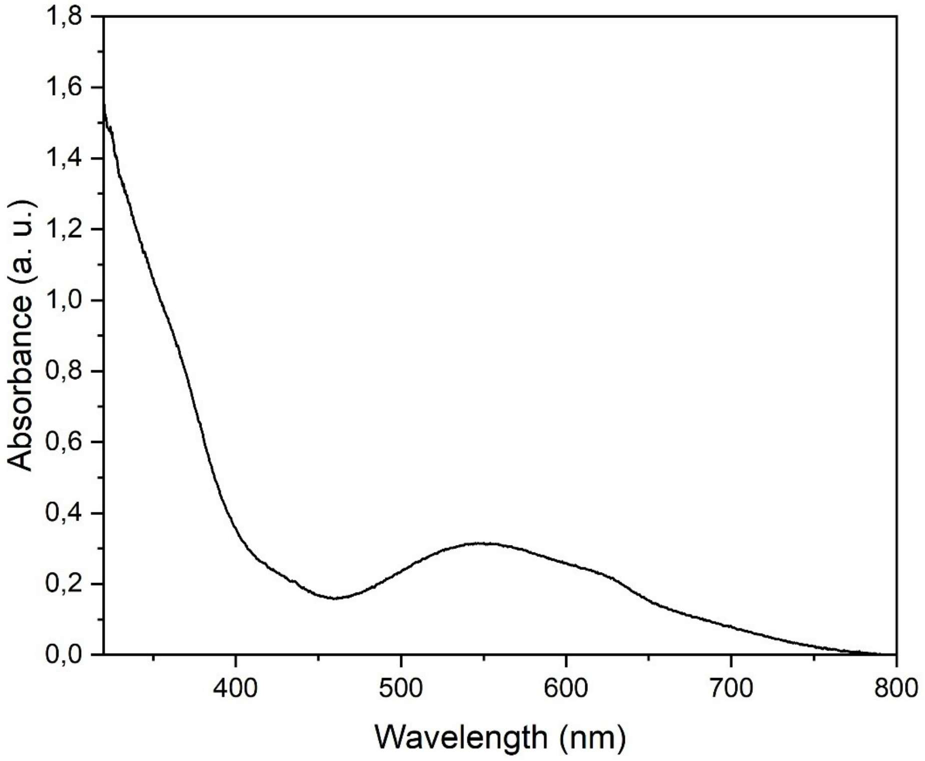

Figure S8. UV/Vis spectrum of a $5x10^{-4}$ M solution of **3b** in toluene/PhF (3/1) at ambient temperature.

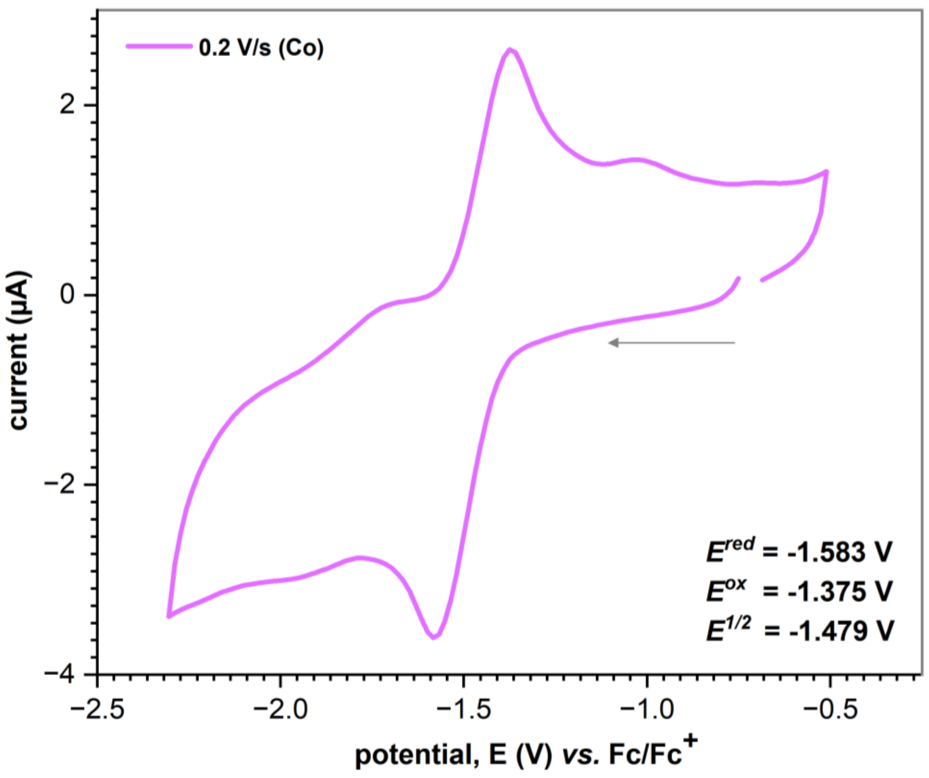

Figure S9. Cyclic voltammogram of a 3 mM solution of **3a** in THF under Ar (*i.e.* in a glove box); 0.1 M [^{n}Bu$_4$N][PF$_6$]; 0.2 V·s^{-1}.

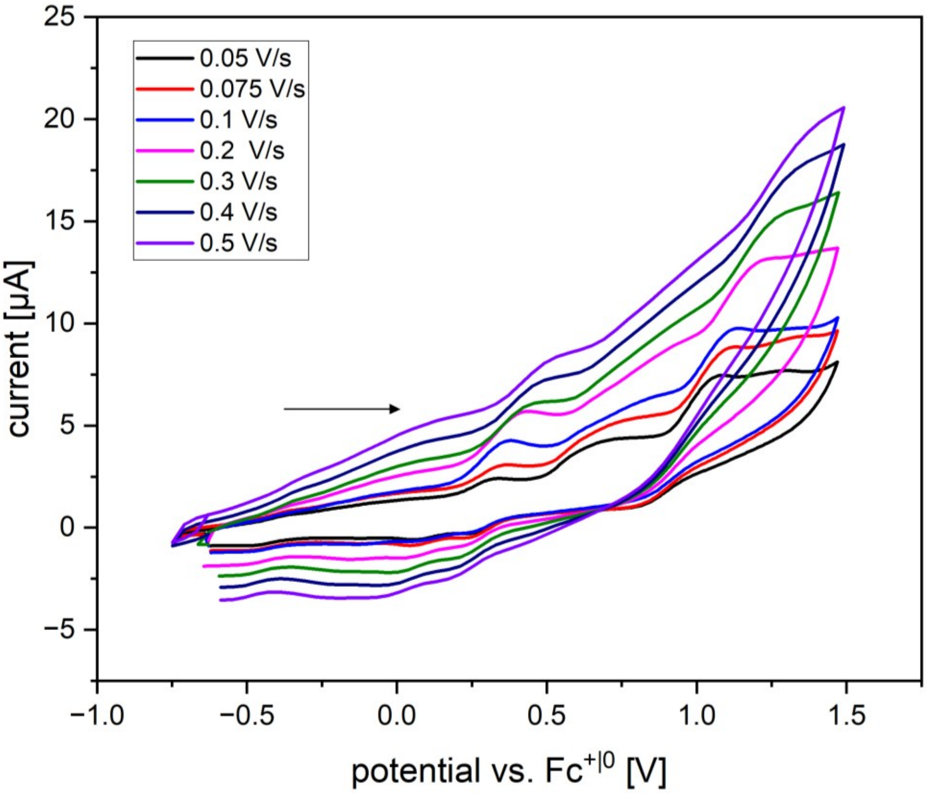

Figure S10. Stack-plot of oxidative cyclic voltammograms of a 3 mM solution of **3a** in THF under Ar (*i.e.* in a glove box); 0.1 M [^{n}Bu$_4$N][PF$_6$], wit varying scan rate.

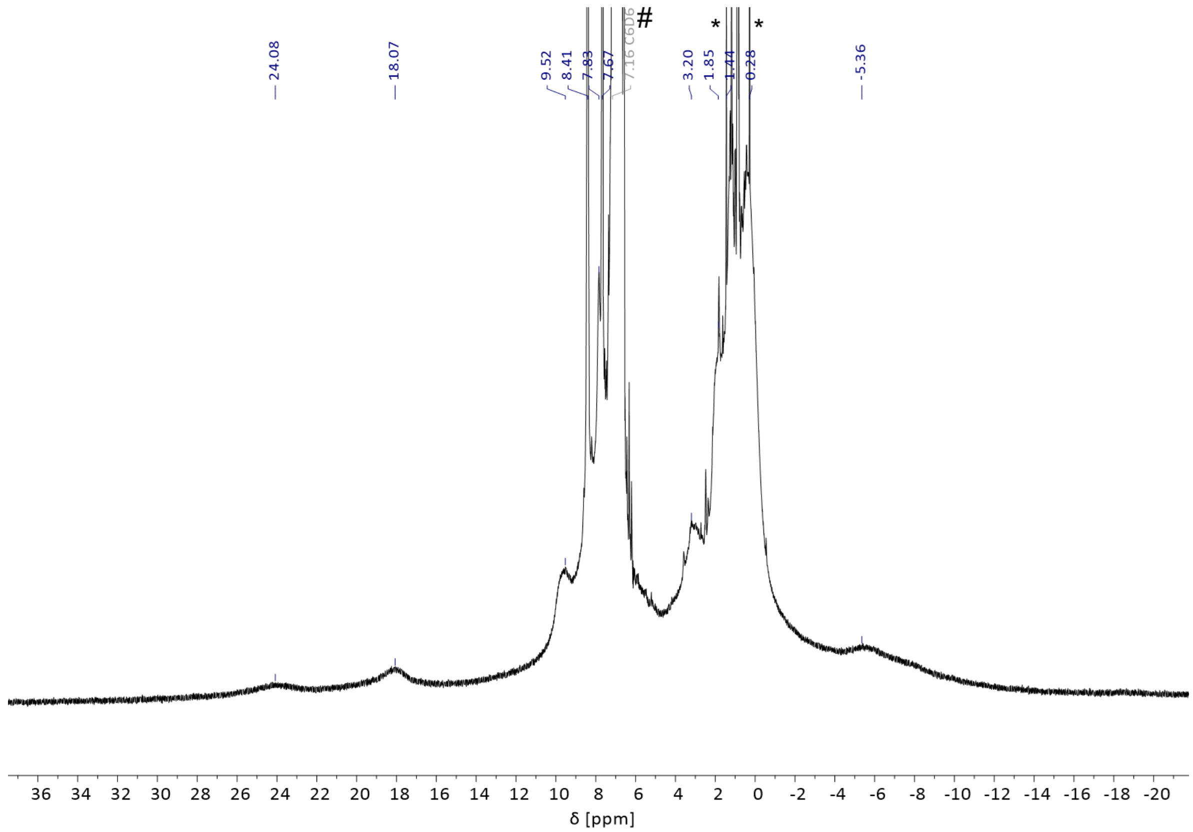

Figure S11. ^{1}H NMR spectrum of **4a** in a C_6D_6/PhF (3:1) solution. # marks PhF, while * marks small amounts of residual pentane.

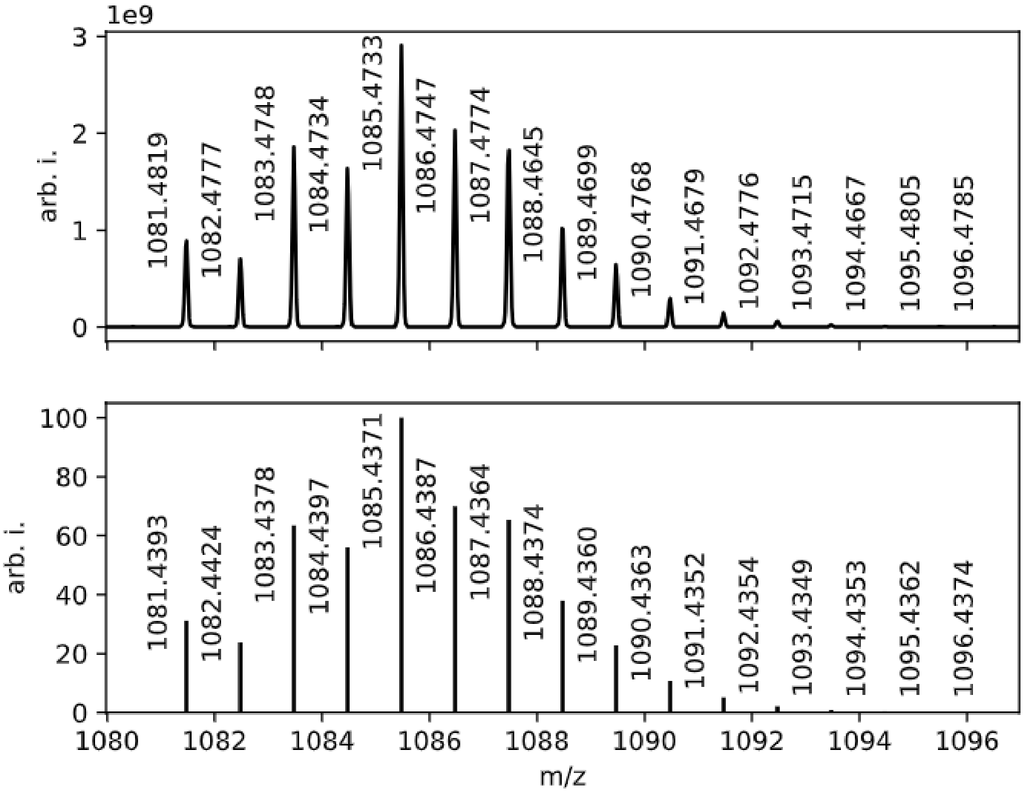

Figure S12. Top: Cutout from LIFDI/MS of **4a**; Bottom: Calculated MS spectrum of $[M-BAr_4^F]^+$.

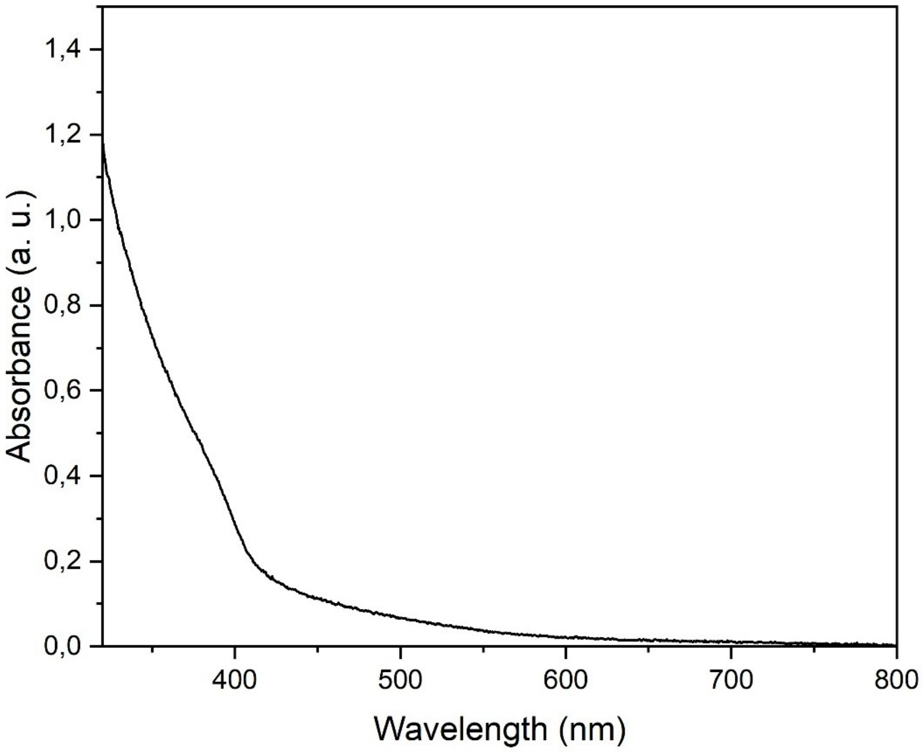

Figure S13. UV/Vis spectrum of a 2.5x10^{-4} M solution of **4a** in toluene/PhF (3/1) at ambient temperature.

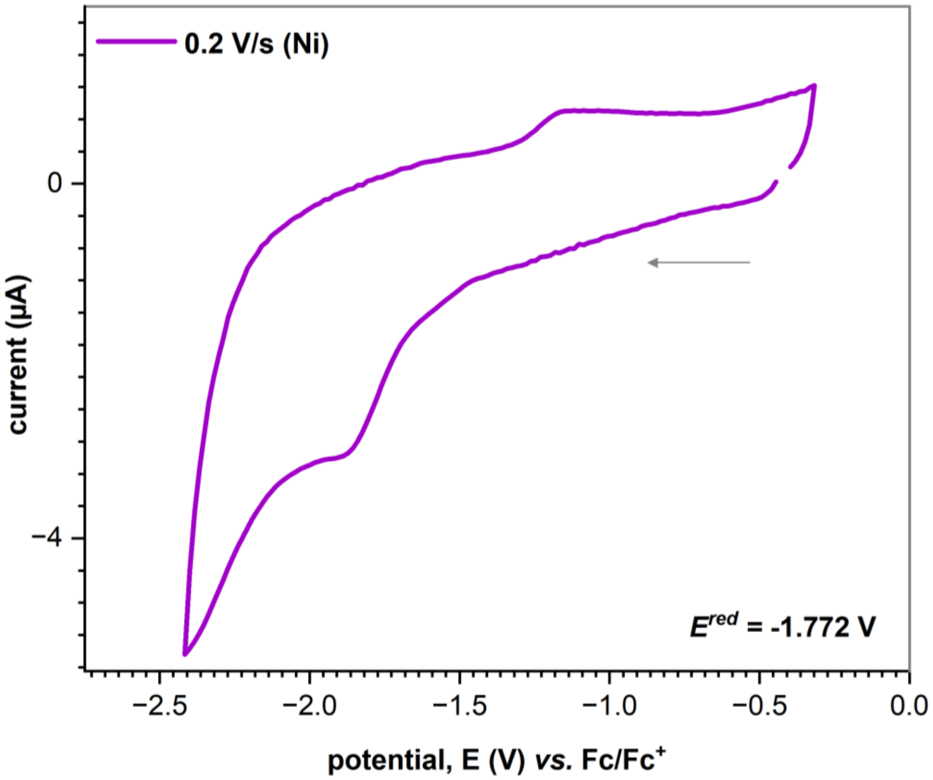

Figure S14. Cyclic voltammogram of a 3 mM solution of **4a** in THF under Ar (*i.e.* in a glove box); 0.1 M [^{n}Bu$_4$N][PF$_6$]; 0.2 V·s^{-1}.

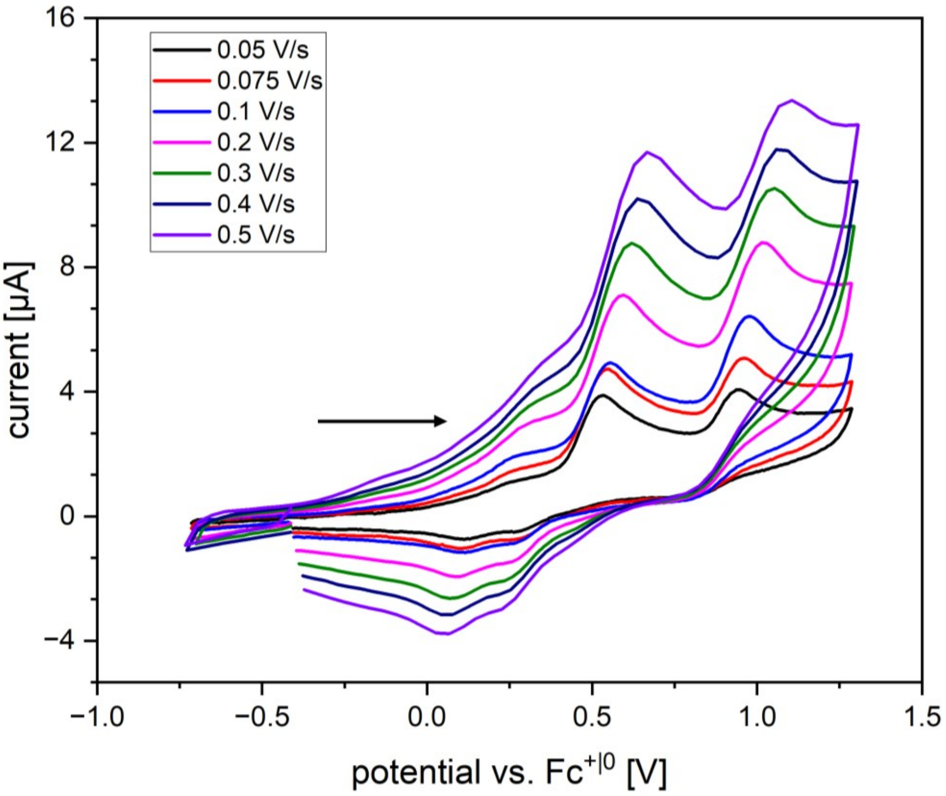

Figure S15. Stack-plot of oxidative cyclic voltammograms of a 3 mM solution of **4a** in THF under Ar (*i.e.* in a glove box); 0.1 M [$^{n}Bu_4N$][PF_6], wit varying scan rate.

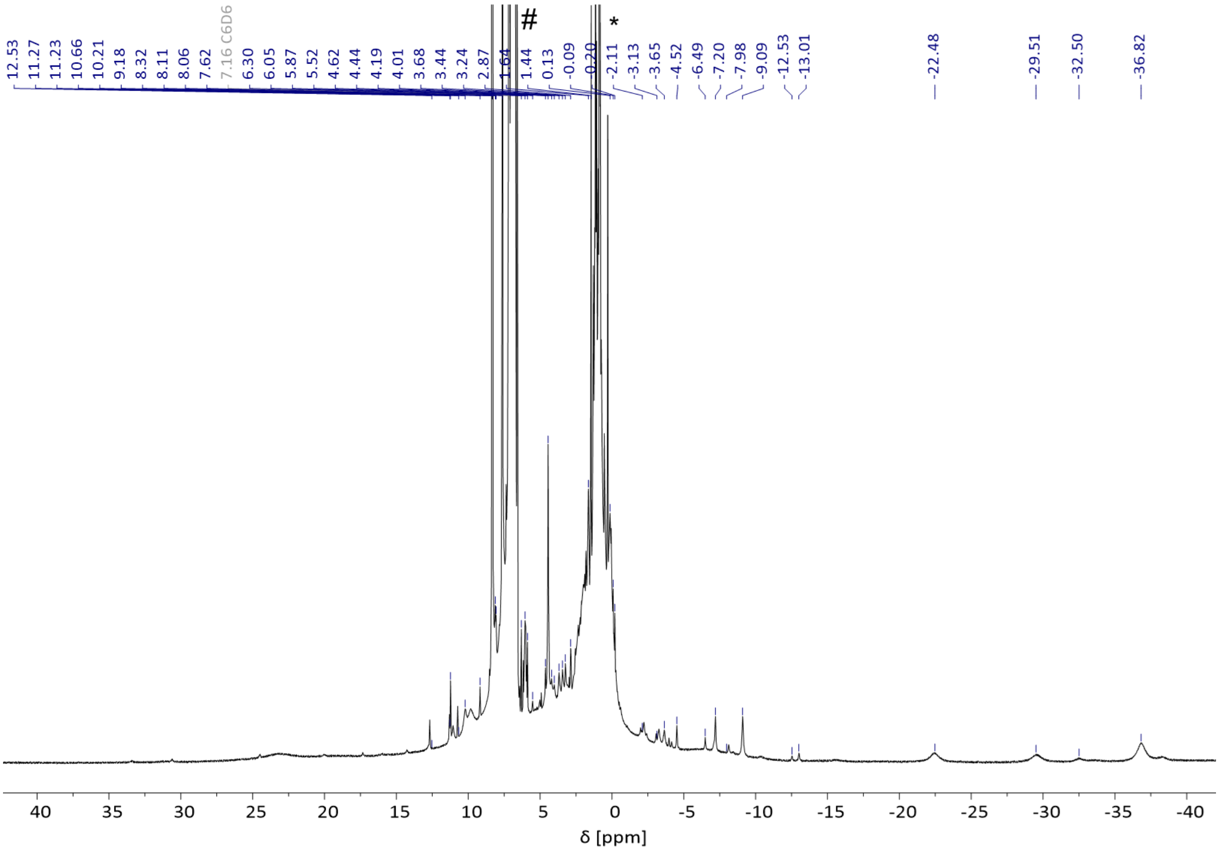

Figure S16. ^{1}H NMR spectrum of **4b** in a C_6D_6/PhF (3:1) solution. # marks PhF, while * marks small amounts of residual pentane.

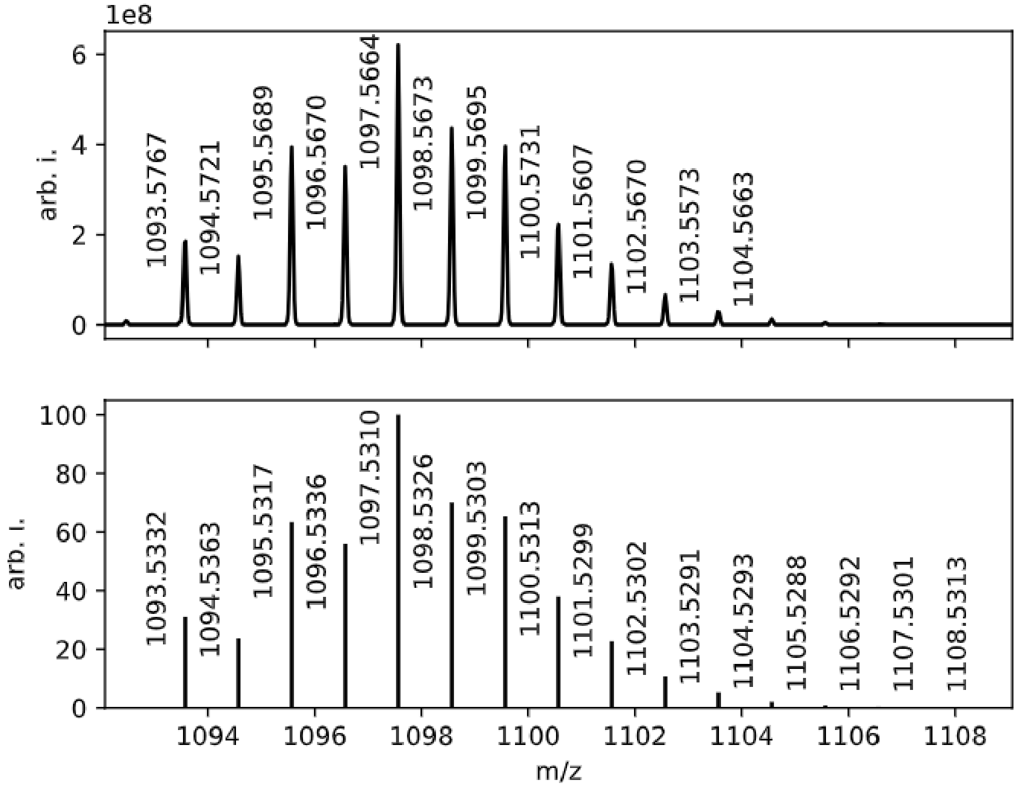

Figure S17. Top: Cutout from LIFDI/MS of **4b**; Bottom: Calculated MS spectrum of $[M\text{-}BAr_4^F]^+$.

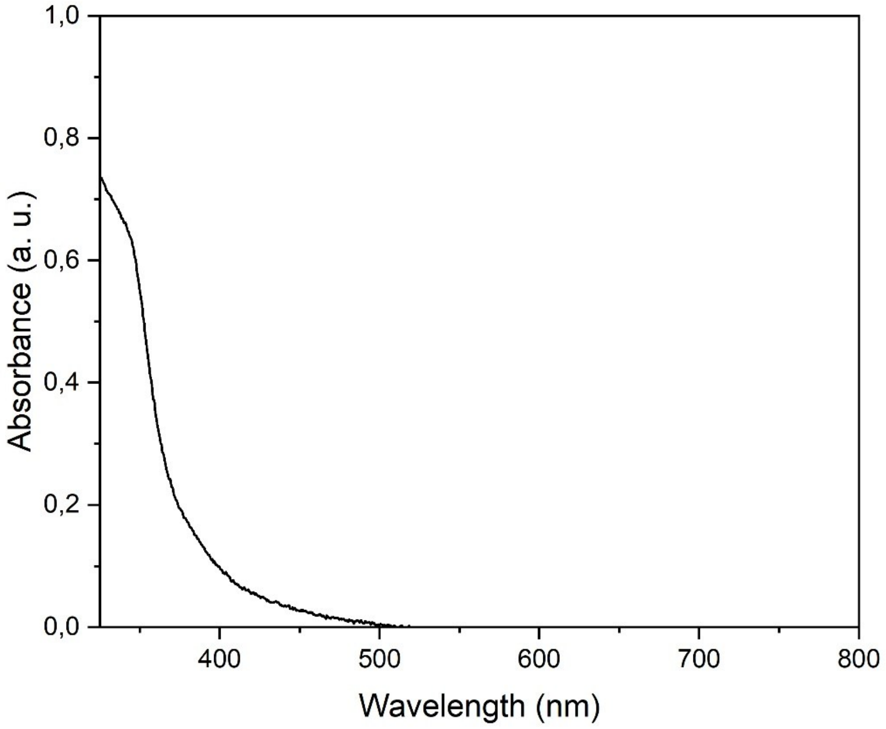

Figure S18. UV/Vis spectrum of a $1.25x10^{-4}$ M solution of **4b** in toluene/PhF (3/1) at ambient temperature.

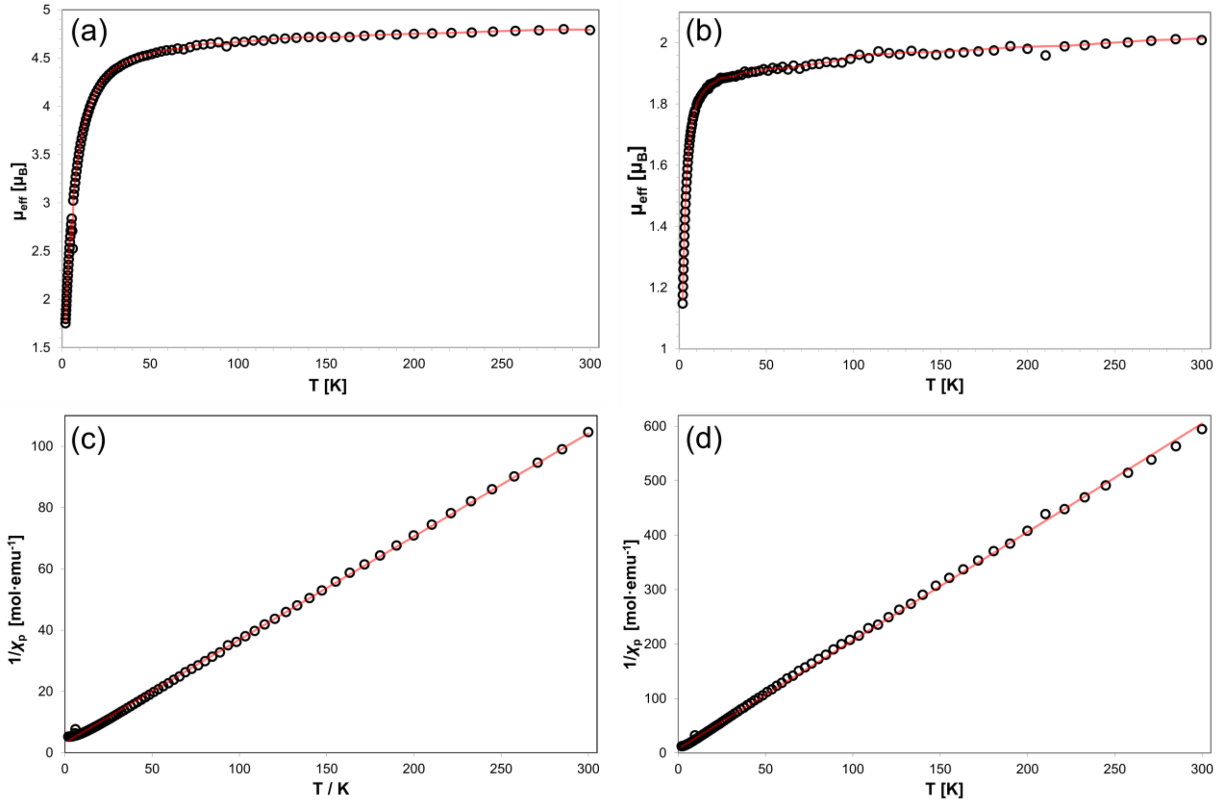

Figure S19. Plots of μ_{eff} *vs.* T for (a) **2** and (b) **4a**, and of $1/\chi_p$ *vs.* T for (c) **2** and (d) **4a**.

Alkene hydrogenation catalysed by complexes 2-4.

To probe their reactivity as potential catalysts, compounds [IPr·M(η^6-tol)][BAr$^F{}_4$] and **2-4** were screened in hydrogenation catalysis. This aims to directly compare with our earlier work, *i.e.* utilising closely related M^0 complexes,[1] and as such mimicked conditions employed there (20h, 60 °C, 1.5 bar H_2) for the hydrogenation of vinyltrimethylsilane and cyclopentene. Vinyltrimethylsilane represents a primary alkene that cannot undergo isomerisation, and is easily traceable by ^{1}H NMR spectroscopy; cyclopentene is similarly traceable, but has been seen to undergo polymerisation under the given conditions. Its inclusion here aimed to define the selectivity of these M^I catalyst systems. The results are summarised in Table 1.

For iron(I) complexes [IPr·Fe(η^6-tol)][BAr$^F{}_4$] and **2,** no formation of the expected hydrogenation products was observed after 20h of reaction. The lack of activity may seem surprising, considering various iron complexes in literature which are effective alkene hydrogenation catalysts.[39-40] However, we note that no examples of iron(I) species are reported, to the best of our knowledge, which are active in olefin

Table 1. Catalytic alkene hydrogenation utilising transition metal-based catalysts [IPr·M(η[6]-tol)][BAr$^F_4$] and **2-4**.[a]

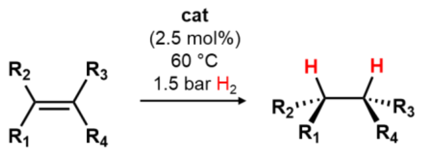

entry	catalyst	substrate	product	yield (%)[b]
1	[IPr·Fe(η[6]-tol)]+			0
2	**2**			0
3	[IPr·Co(η[6]-tol)]+			12
4	**3a**	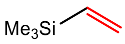	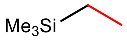	6
5	**3b**			<5
6	[IPr·Ni(η[6]-tol)]+			31
7	**4a**			35
8	**4b**			46
9	[IPr·Fe(η[6]-tol)]+			0
10	**2**			0
11	[IPr·Co(η[6]-tol)]+			<5
12	**3a**	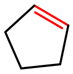	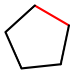	<5
13	**3b**			<5
14	[IPr·Ni(η[6]-tol)]+			0
15	**4a**			<5
16	**4b**			11

[a] Conducted in gas-tight pressure flasks, as solutions in 0.4 mL (3:1 C_6D_6/PhF), 2.5 mol% cat., 60 °C, 1.5 bar H_2, and 0.022 mmol mesitylene as an internal standard. [b] All values are determined by integration of ^{1}H NMR spectra.

hydrogenation; rather, catalytically competent systems typically involve Fe^{II} or Fe^0 species.[2],[3] Moving to Co^I species made little difference, affording little more than 10% conversion of vinyltrimethylsilane, and less than 5% of cyclopentane under the given catalytic conditions, despite Co^I complexes being known as competent alkene hydrogenation catalysts.[4],[5]

For nickel, moderate catalytic activity was observed; **4b** proved the most active nickel complex, affording 46% yield of ethyltrimethylsilane under the given conditions. However, the observed complete consumption of alkene substrate indicates the formation of multiple side products, as evidenced by additional resonances in the aliphatic region of the ^{1}H NMR spectrum. We observe a similar effect for cyclopentene hydrogenation, with up to 11% yield of cyclopentane, but full substrate consumption

points to competing side reactions, most likely polymerisation, which is established for Ni catalysts including in our earlier work.[1] Taken as a whole, the given M^{I}-complexes are poor hydrogenation catalysts, in contrast to numerous TM^{0} systems which have been reported.

General Method of Catalytic Hydrogenation.

0.4 mL of a freshly prepared 4 mM stock solution of **2**, **3**, or **4** or [IPr·M(η^6-tol)][BAr^F_4] (2.5 mol%) in C_6D_6/PhF (3/1) with 0.022 mmol mesitylene as internal standard was placed in a thick-walled Teflon-sealed Schlenk flask. The alkenes (0.064 mmol) were subsequently added in the form of a freshly prepared stock solution in C_6D_6 *via* micro-pipette. Hydrogen (1.5 bar) was added through one freeze-pump-thaw cycle of the respective sample. The resulting solution was heated at 60 ºC, and the reaction was monitored via ^{1}H NMR spectroscopy after 20 hours. The progress was evidenced by the integration of the alkane signals compared to the internal standard in the sample.

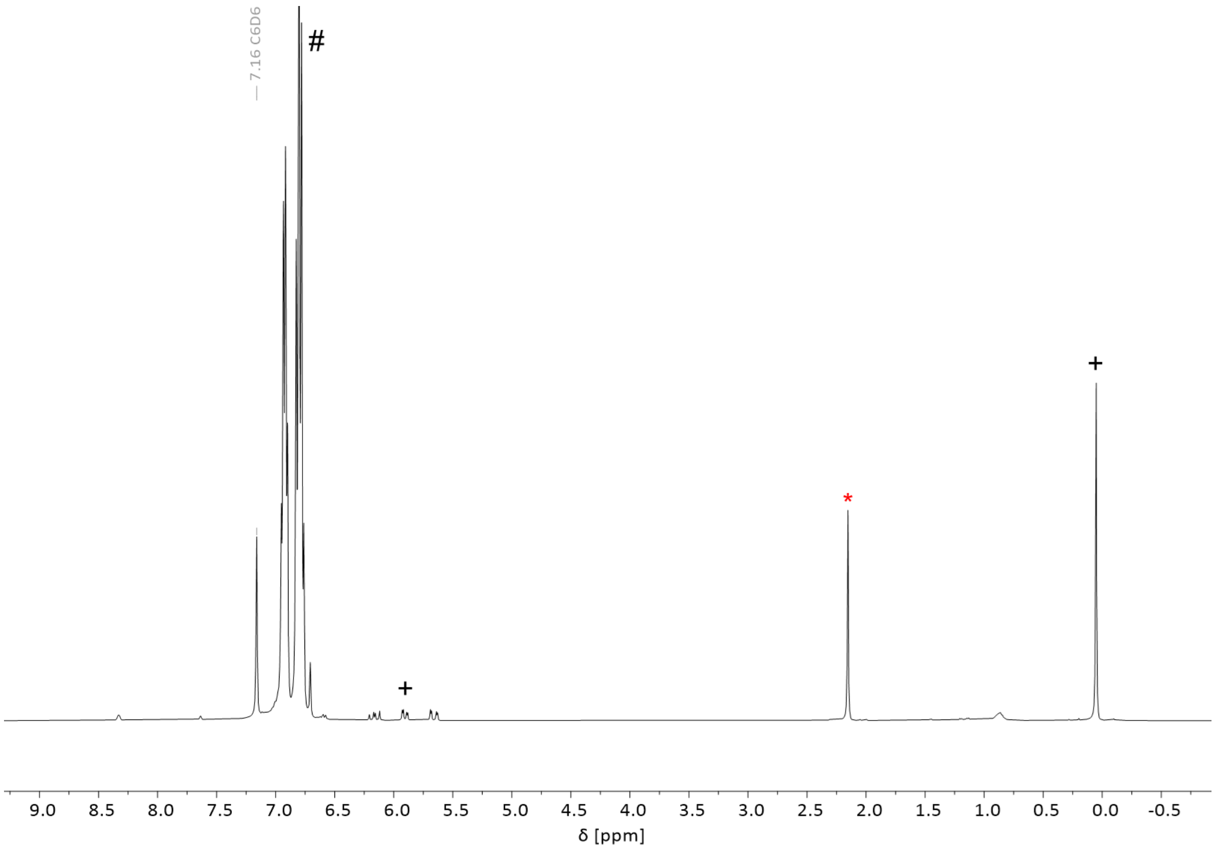

Figure S20. ^{1}H NMR of the reaction mixture of [IPr·Fe(η^6-tol)][BAr^F_4] (2.5 mol%) and vinyltrimethysilane in C_6D_6/PhF (3/1) after 20h reaction time. # marks PhF; + marks vinyltrimethylsilane; * indicates the internal standard mesitylene.

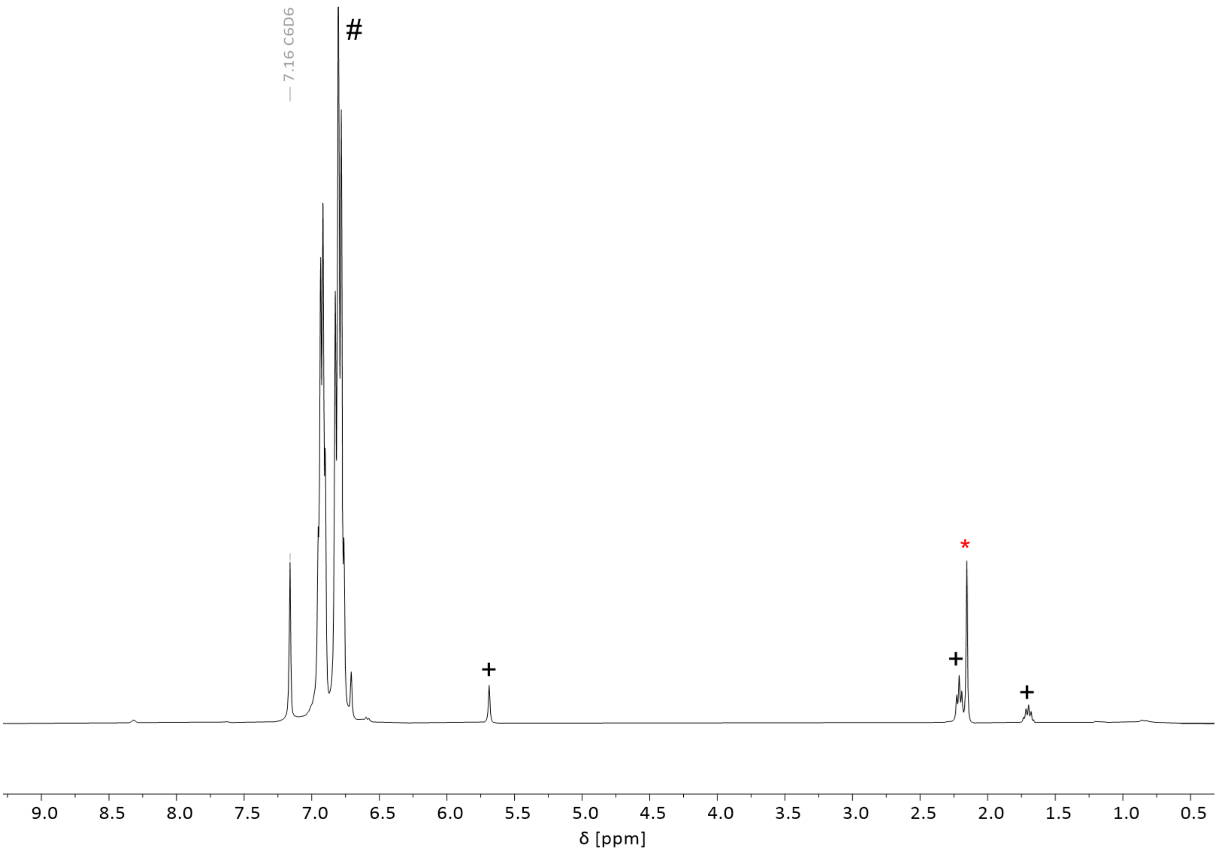

Figure S21. ^{1}H NMR of the reaction mixture of [IPr·Fe(η^6-tol)][BAr$^F{}_4$] (2.5 mol%) and cyclopentene in C_6D_6/PhF (3/1) after 20h reaction time. # marks PhF; + marks cyclopentene; * indicates the internal standard mesitylene.

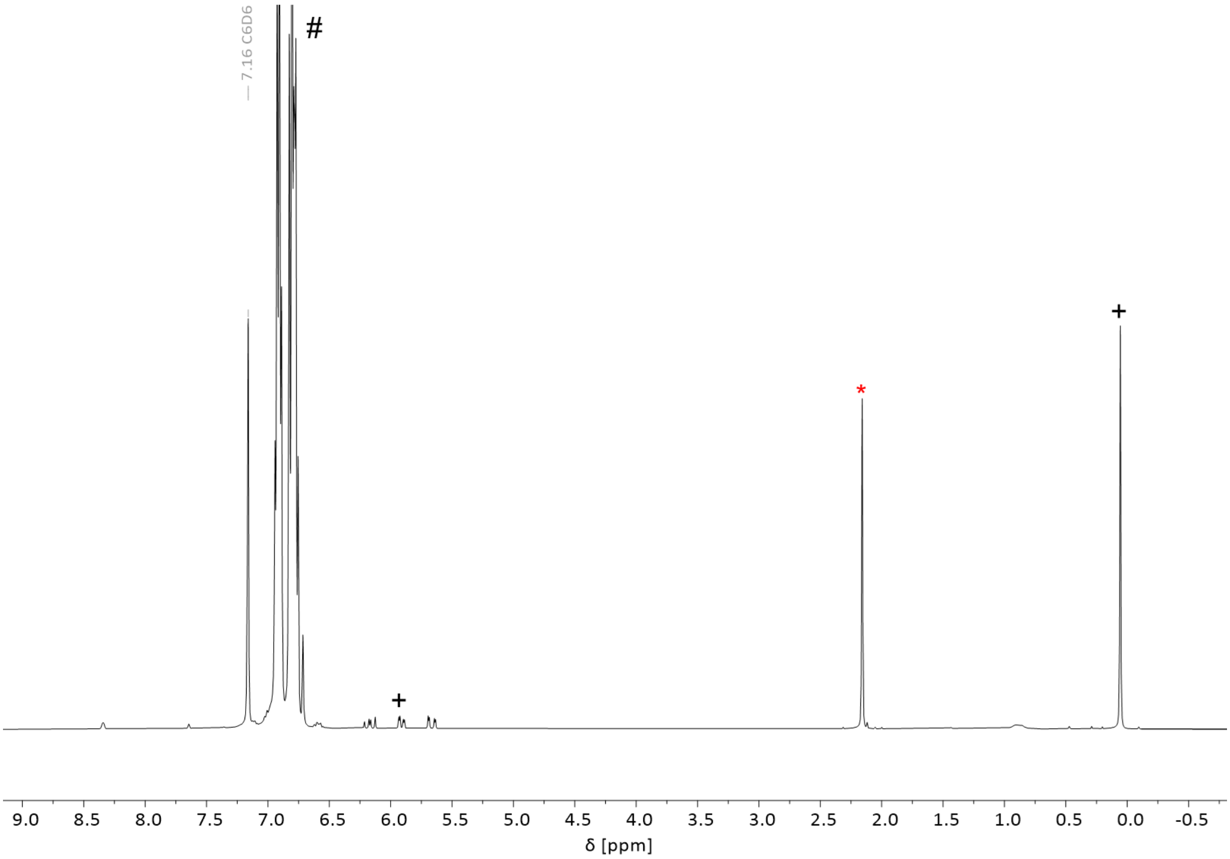

Figure S22. ^{1}H NMR of the reaction mixture of **2** (2.5 mol%) and vinyltrimethysilane in C_6D_6/PhF (3/1) after 20h reaction time. # marks PhF; + marks vinyltrimethylsilane; * indicates the internal standard mesitylene.

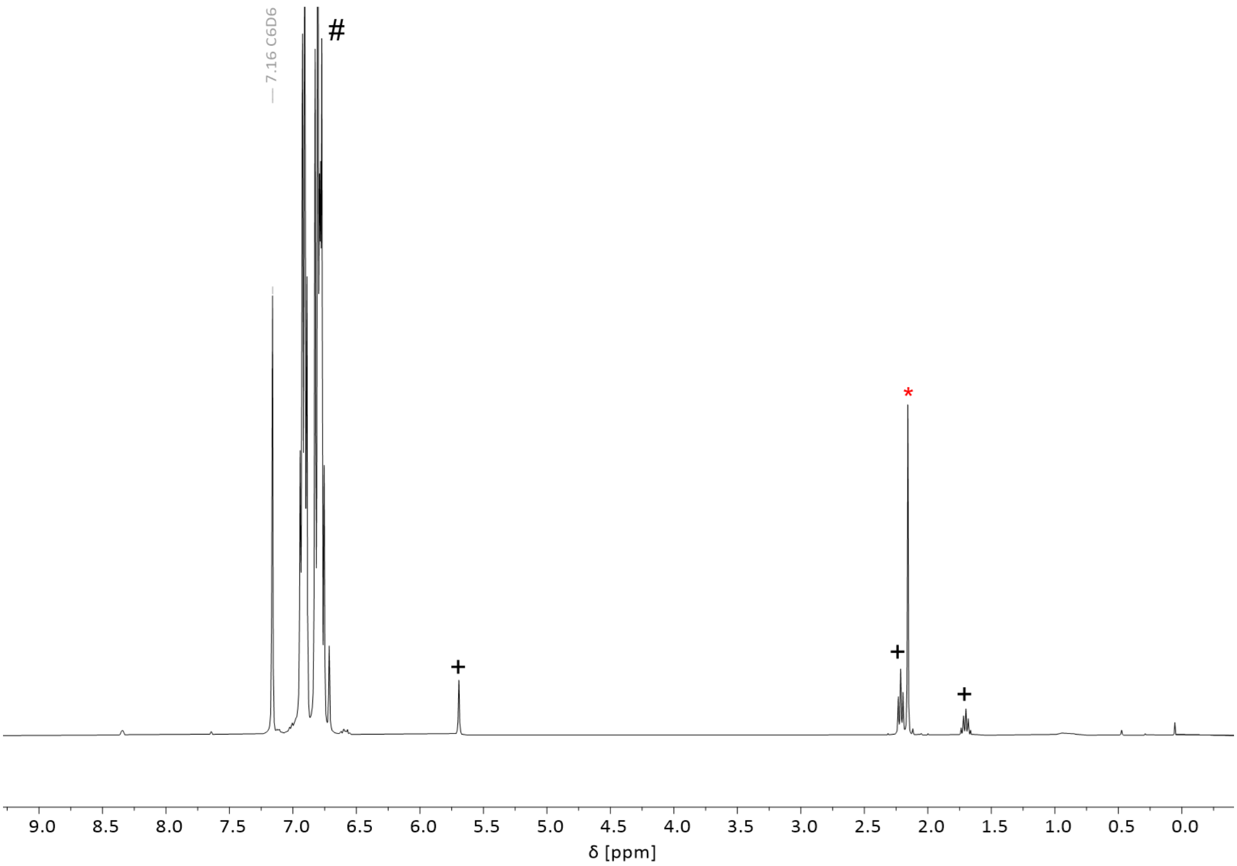

Figure S23. ^{1}H NMR of the reaction mixture of **2** (2.5 mol%) and cyclopentene in C_6D_6/PhF (3/1) after 20h reaction time. # marks PhF; + marks cyclopentene; * indicates the internal standard mesitylene.

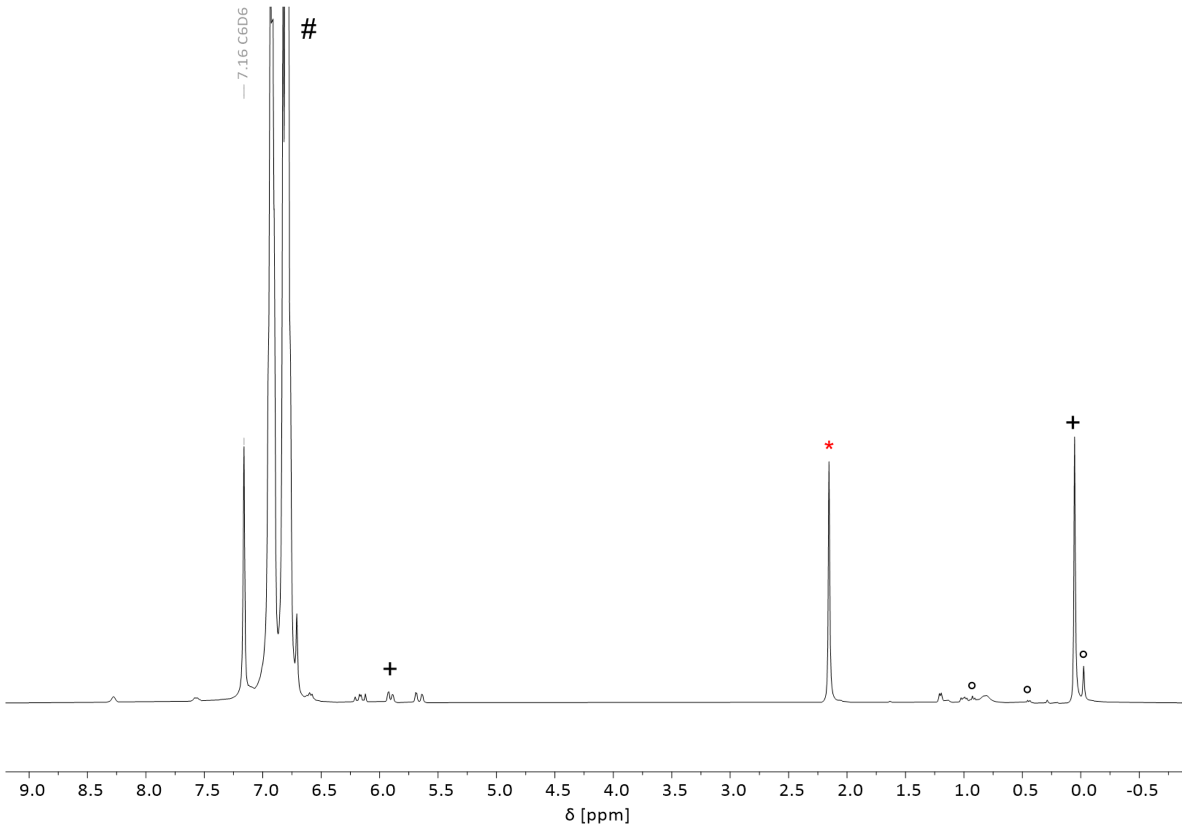

Figure S24. ^{1}H NMR of the reaction mixture of [IPr·Co(η^6-tol)][BAr$^F{}_4$] (2.5 mol%) and vinyltrimethysilane in C_6D_6/PhF (3/1) after 20h reaction time. # marks PhF; + marks vinyltrimethylsilane; ° marks ethyltrimethylsilane; * indicates the internal standard mesitylene.

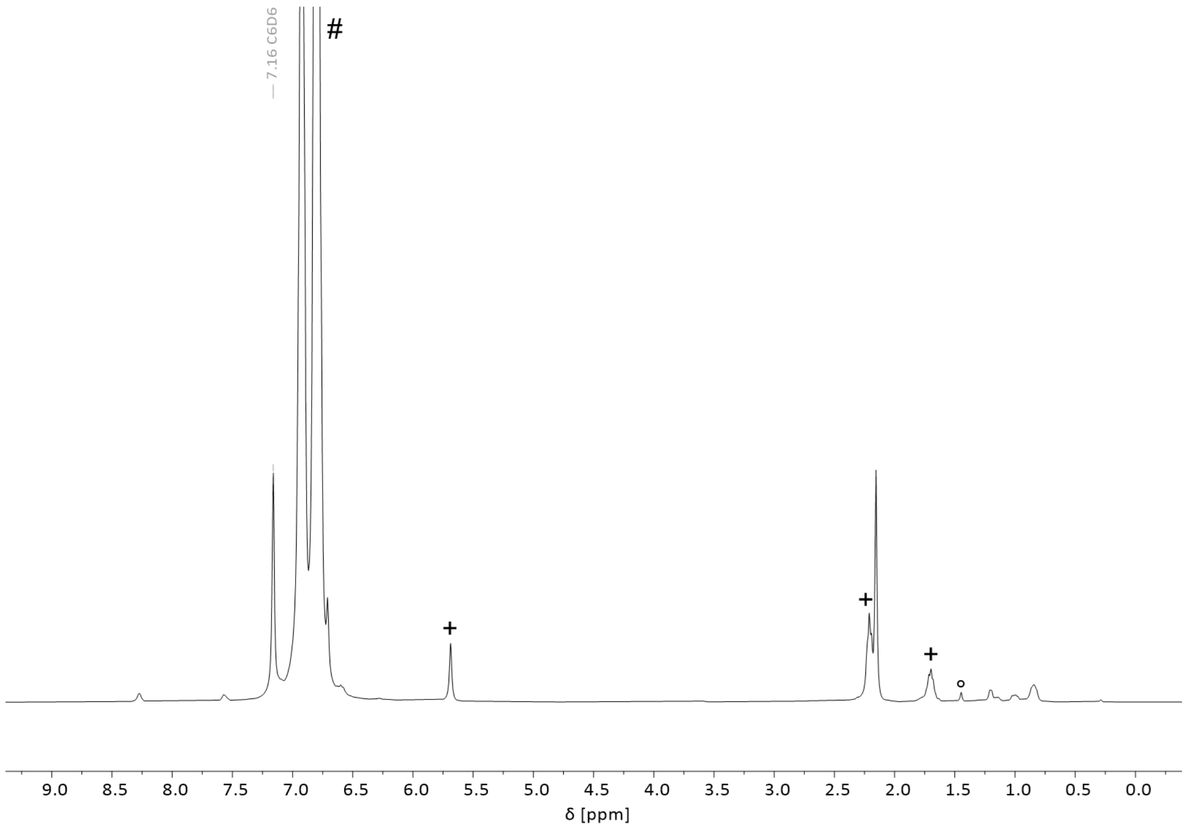

Figure S25. ^{1}H NMR of the reaction mixture of [IPr·Co(η^6-tol)][BAr$^F{}_4$] (2.5 mol%) and cyclopentene in C_6D_6/PhF (3/1) after 20h reaction time. # marks PhF; + marks cyclopentene; ° marks cyclopentane; * indicates the internal standard mesitylene.

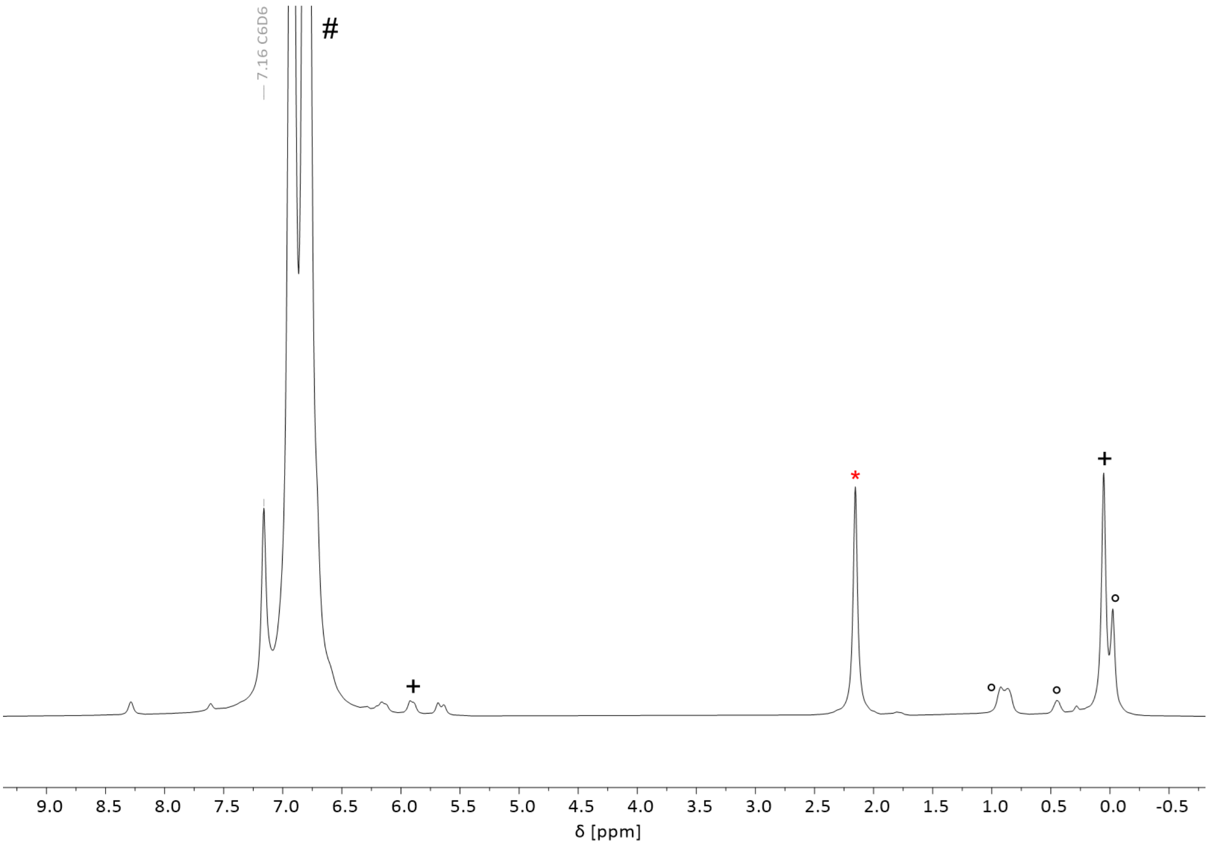

Figure S26. ^{1}H NMR of the reaction mixture of **3a** (2.5 mol%) and vinyltrimethysilane in C_6D_6/PhF (3/1) after 20h reaction time. # marks PhF; + marks vinyltrimethylsilane; ° marks ethyltrimethylsilane; * indicates the internal standard mesitylene.

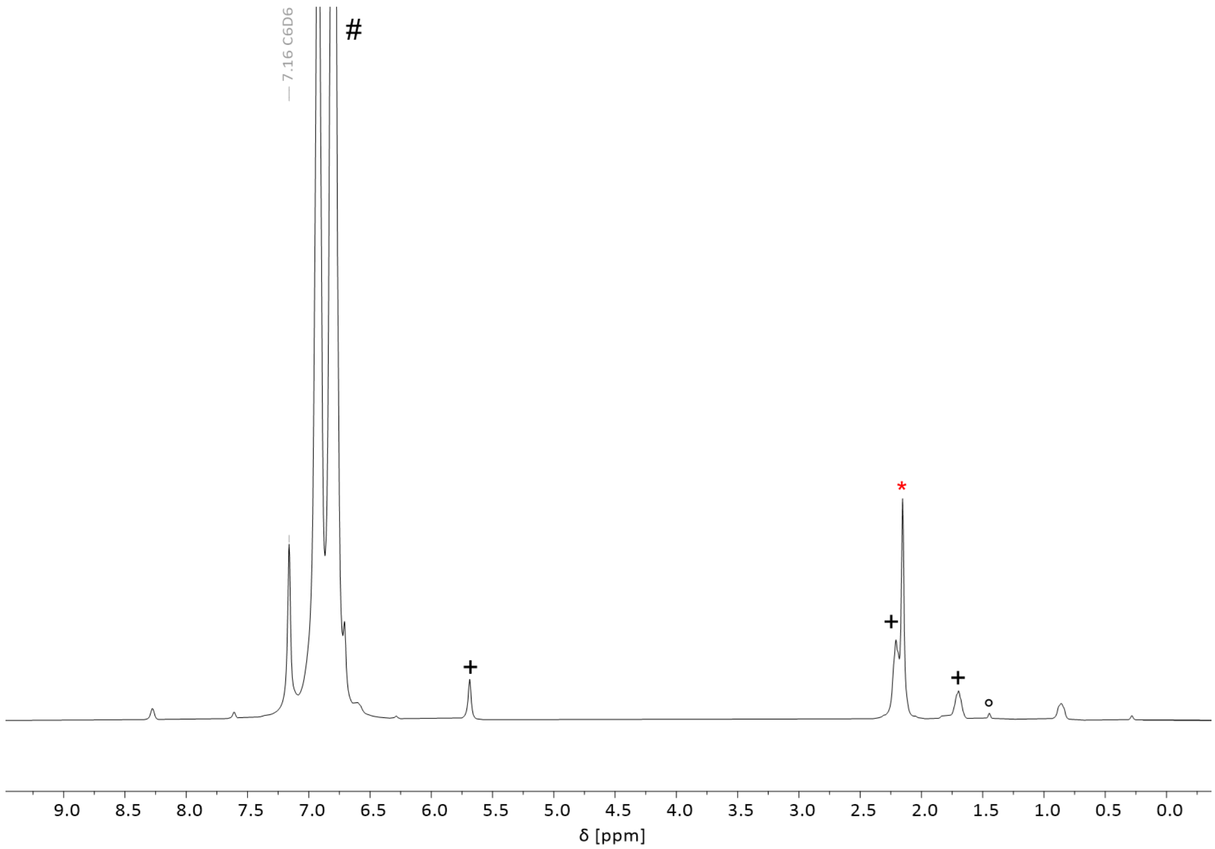

Figure S27. ^{1}H NMR of the reaction mixture of **3a** (2.5 mol%) and cyclopentene in C_6D_6/PhF (3/1) after 20h reaction time. # marks PhF; + marks cyclopentene; ° marks cyclopentane; * indicates the internal standard mesitylene.

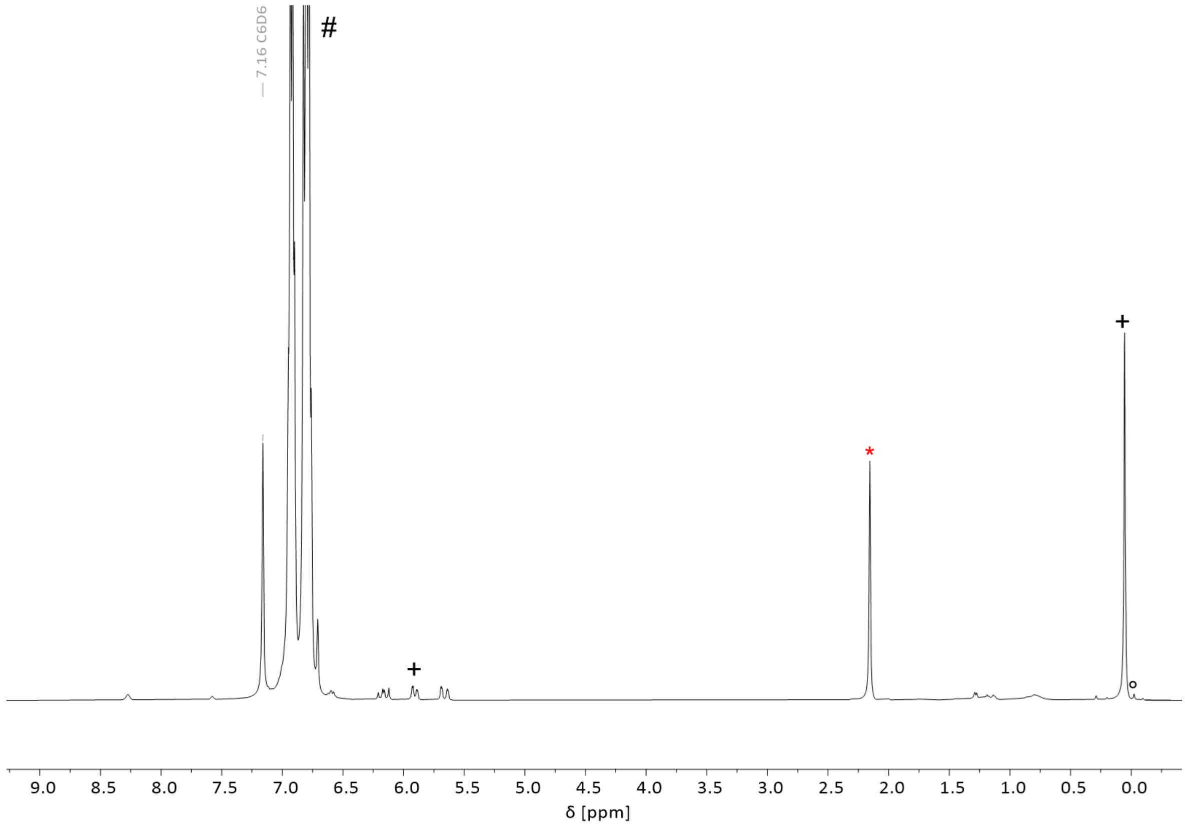

Figure S28. ^{1}H NMR of the reaction mixture of **3b** (2.5 mol%) and vinyltrimethysilane in C_6D_6/PhF (3/1) after 20h reaction time. # marks PhF; + marks vinyltrimethylsilane; ° marks ethyltrimethylsilane; * indicates the internal standard mesitylene.

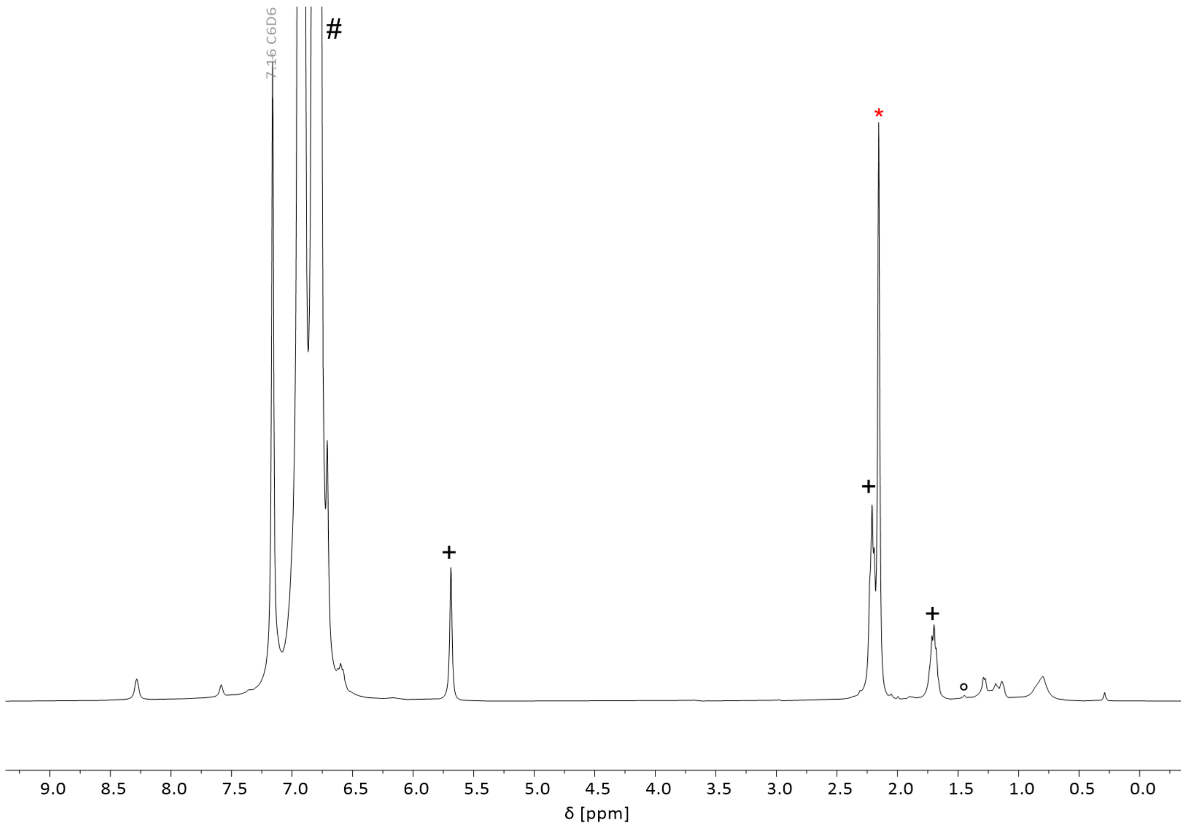

Figure S29. ^{1}H NMR of the reaction mixture of **3b** (2.5 mol%) and cyclopentene in C_6D_6/PhF (3/1) after 20h reaction time. # marks PhF; + marks cyclopentene; ° marks cyclopentane; * indicates the internal standard mesitylene.

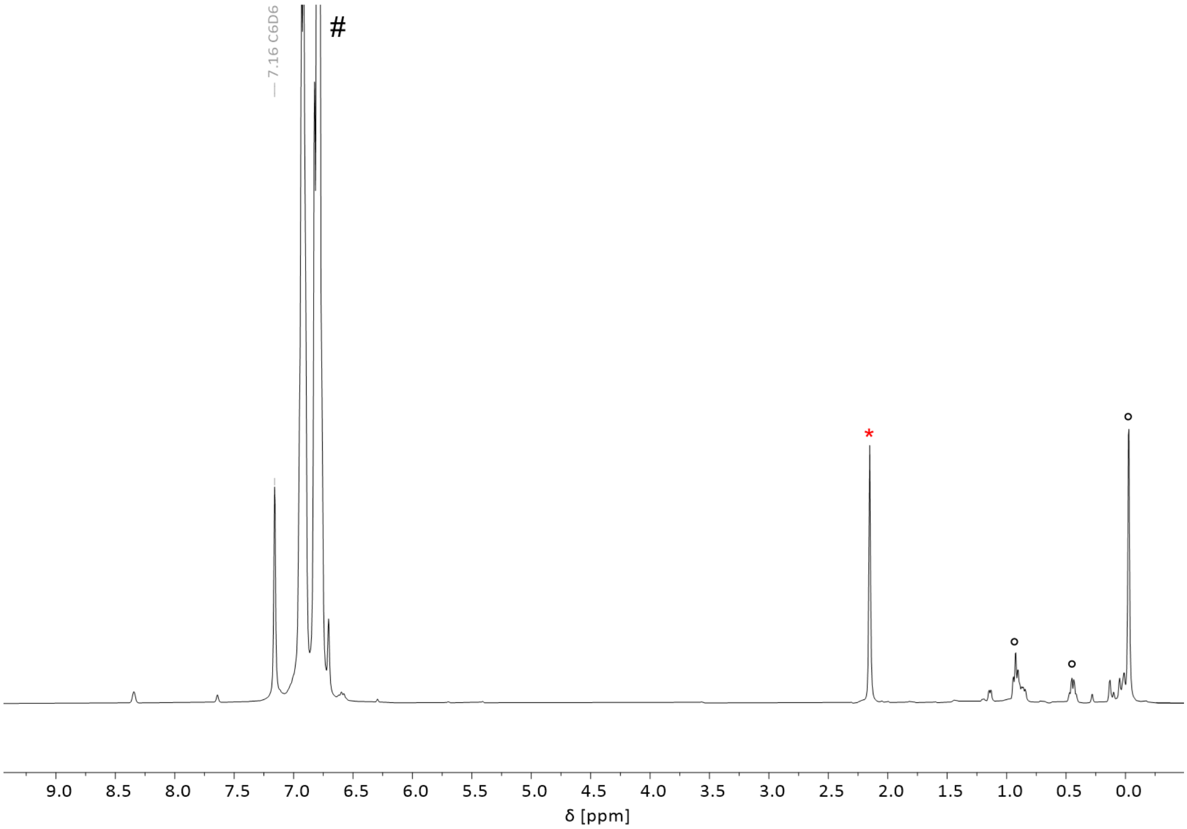

Figure S30. ^{1}H NMR of the reaction mixture of [IPr·Ni(η^6-tol)][$BAr^F{}_4$] (2.5 mol%) and vinyltrimethysilane in C_6D_6/PhF (3/1) after 20h reaction time. # marks PhF; + marks vinyltrimethylsilane; ° marks ethyltrimethylsilane; * indicates the internal standard mesitylene.

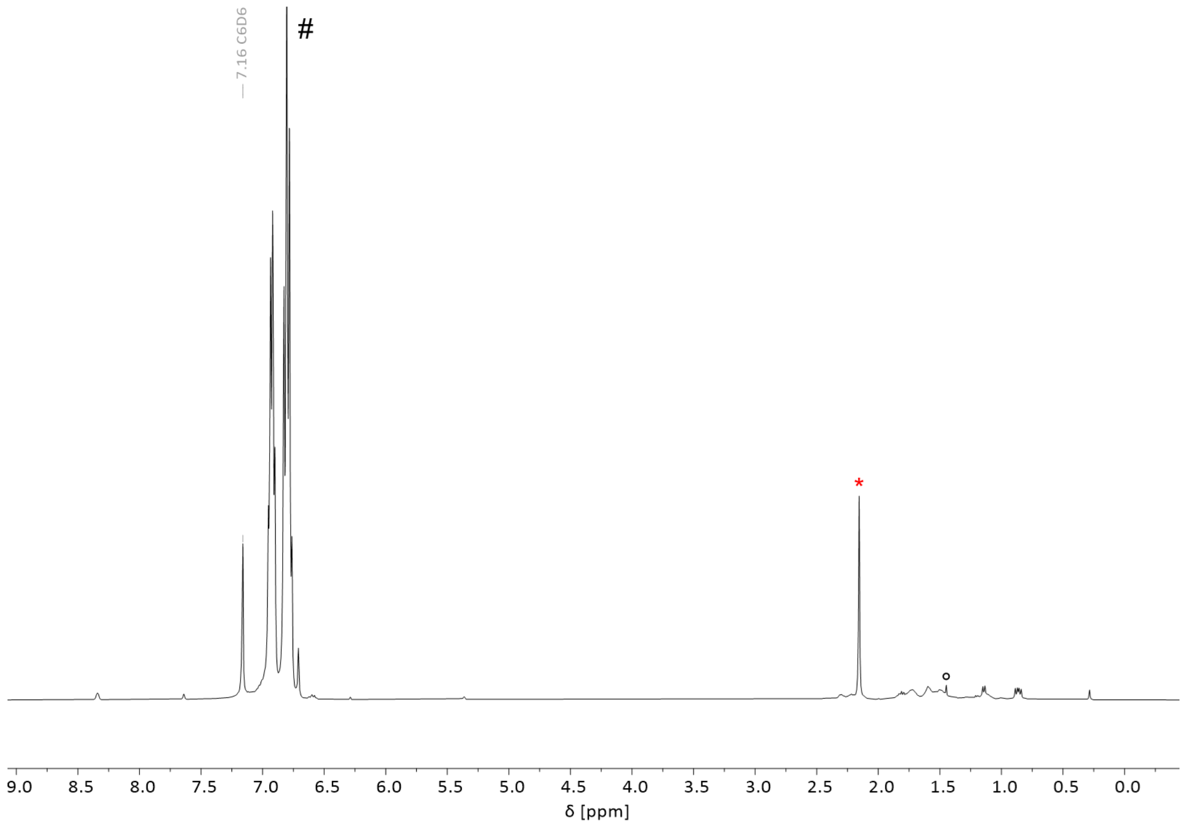

Figure S31. ^{1}H NMR of the reaction mixture of [IPr·Ni(η^6-tol)][BAr$^F{}_4$] (2.5 mol%) and cyclopentene in C_6D_6/PhF (3/1) after 20h reaction time. # marks PhF; + marks cyclopentene; ° marks cyclopentane; * indicates the internal standard mesitylene.

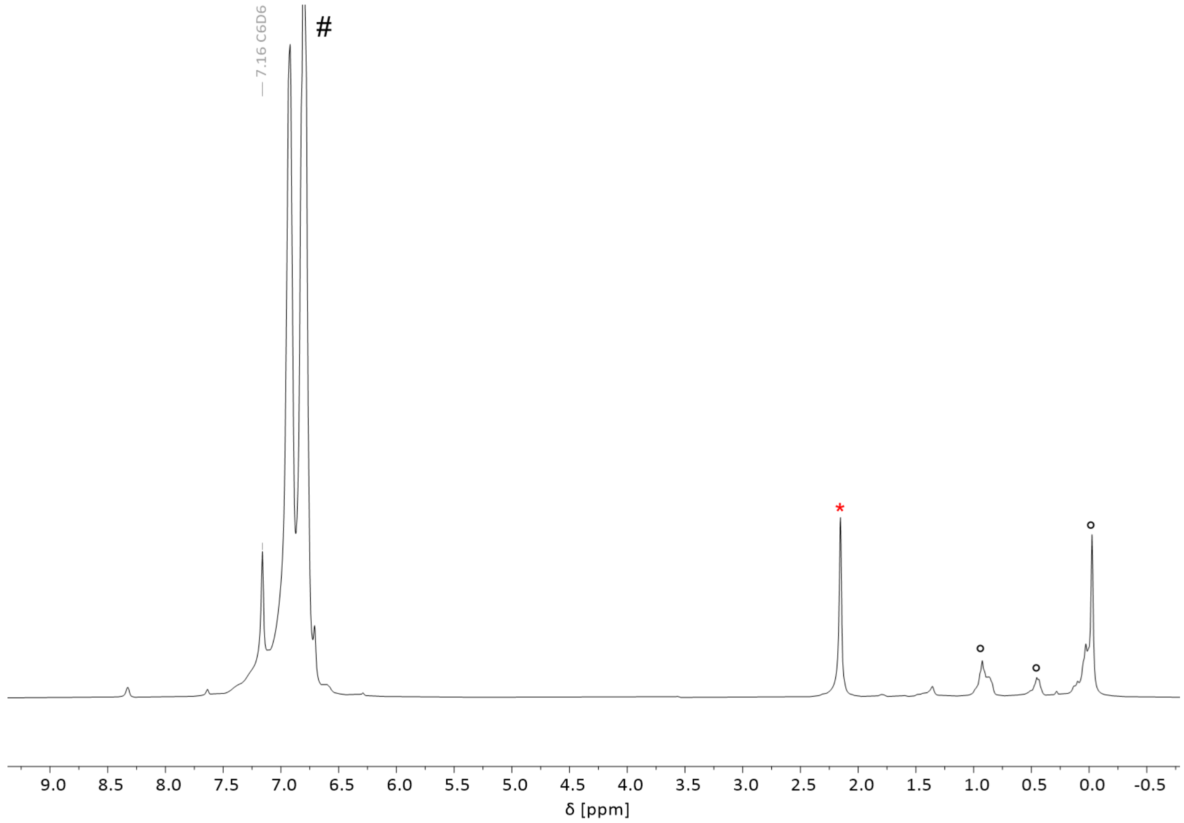

Figure S32. ^{1}H NMR of the reaction mixture of **4a** (2.5 mol%) and vinyltrimethysilane in C_6D_6/PhF (3/1) after 20h reaction time. # marks PhF; + marks vinyltrimethylsilane; ° marks ethyltrimethylsilane; * indicates the internal standard mesitylene.

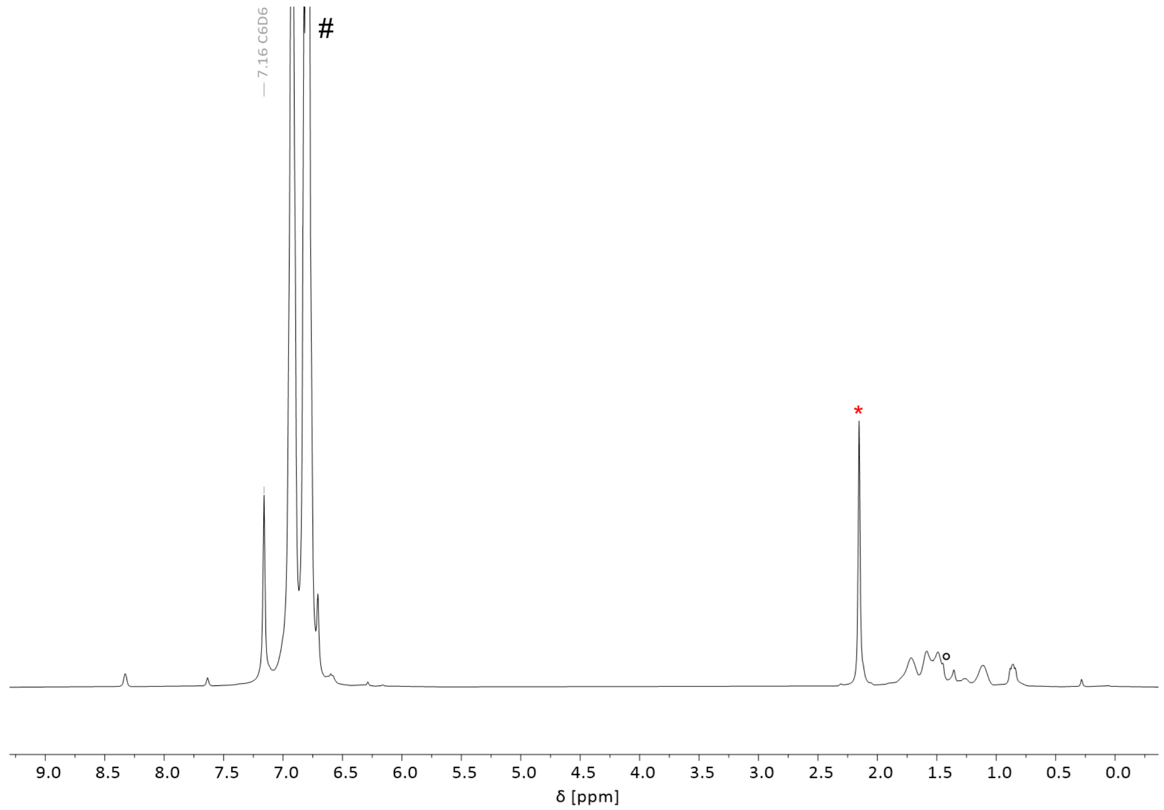

Figure S33. ^{1}H NMR of the reaction mixture of **4a** (2.5 mol%) and cyclopentene in C_6D_6/PhF (3/1) after 20h reaction time. # marks PhF; + marks cyclopentene; ° marks cyclopentane; * indicates the internal standard mesitylene.

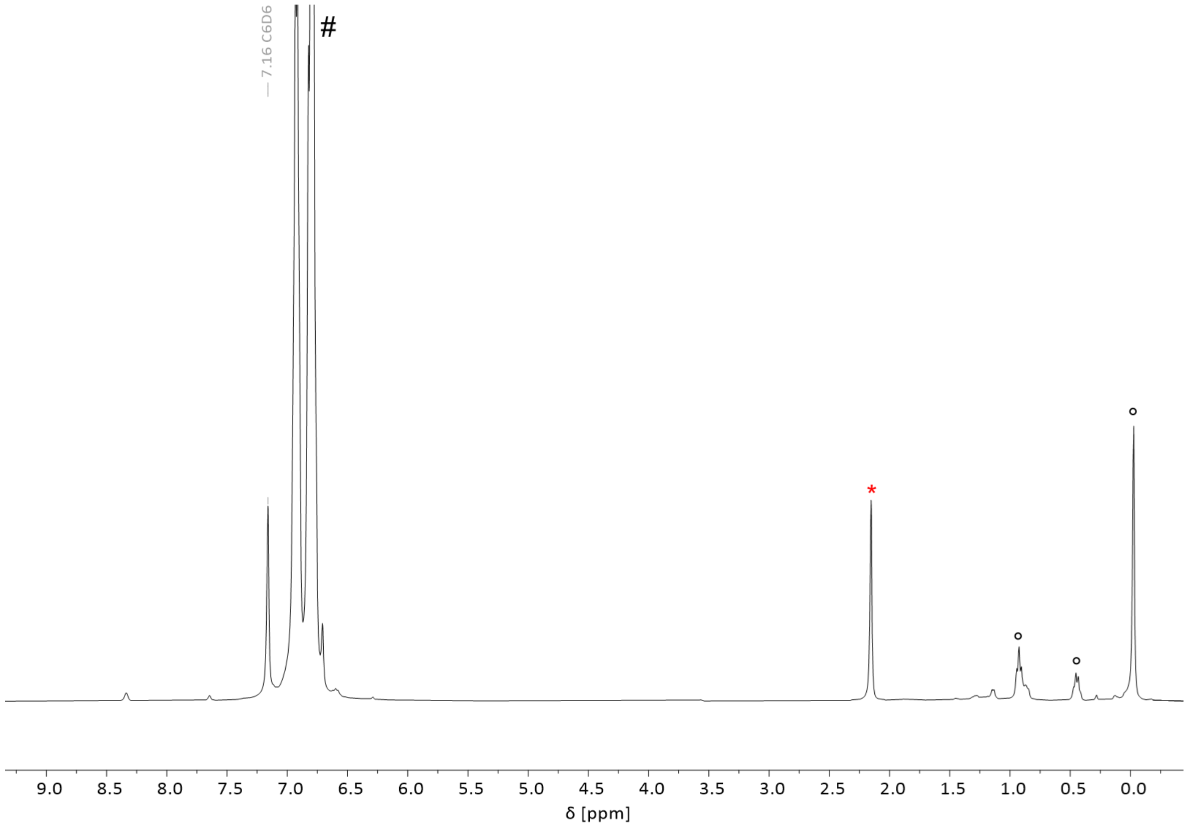

Figure S34. ^{1}H NMR of the reaction mixture of **4b** (2.5 mol%) and vinyltrimethysilane in C_6D_6/PhF (3/1) after 20h reaction time. # marks PhF; + marks vinyltrimethylsilane; ° marks ethyltrimethylsilane; * indicates the internal standard mesitylene.

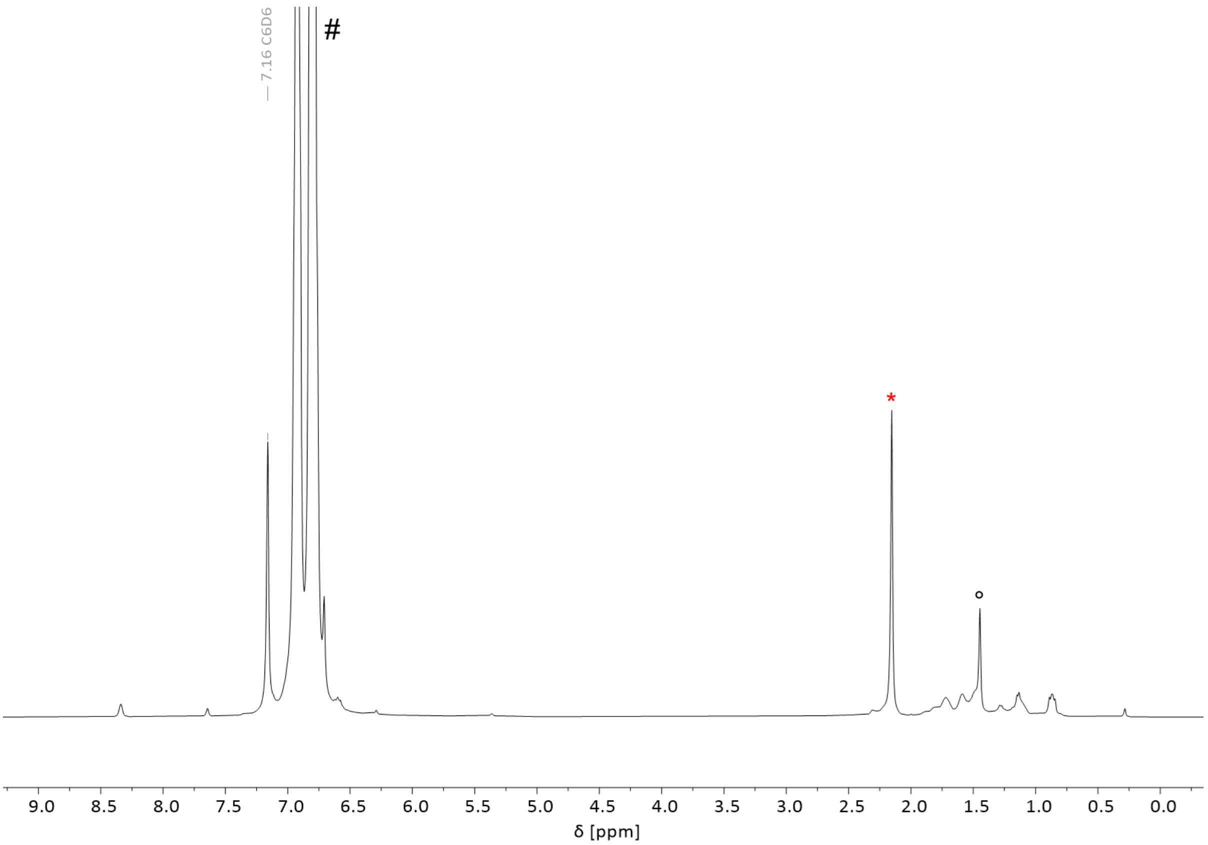

Figure S35. ^{1}H NMR of the reaction mixture of **4a** (2.5 mol%) and cyclopentene in C_6D_6/PhF (3/1) after 20h reaction time. # marks PhF; + marks cyclopentene; ° marks cyclopentane; * indicates the internal standard mesitylene.

2. X-ray crystallographic details

Single crystals of **2**, **3a**, **4a**, and **4b** suitable for X-ray structural analysis were mounted in perfluoroalkyl ether oil on a nylon loop and positioned in a 150 K cold N_2 gas stream. Data collection was performed with a STOE StadiVari diffractometer (MoKα radiation) equipped with a DECTRIS PILATUS 300K detector. Structures were solved by Direct Methods (SHELXS-97),[6] or using SHELXT-16,[7] and refined by full-matrix least-squares calculations against F^2 (SHELXL-2018).[8] The positions of the hydrogen atoms were calculated and refined using a riding model. All non-hydrogen atoms were treated with anisotropic displacement parameters. Crystal data, details of data collections, and refinements for all structures can be found in their CIF files, which are available free of charge via www.ccdc.cam.ac.uk/data_request/cif, and are summarized in Table S1.

Table S1. Summary of X-ray crystallographic data for **2**, **3a**, **4a**, and **4b**.

	**2**	**3b**	**4a**	**4b**
empirical form.	$C_{64}H_{84}FeGeN_3PSi$, $C_{32}H_{12}BF_{24}$	$C_{64}H_{97}CoGeN_3PSi$, $C_{32}H_{12}BF_{24}$	$C_{64}H_{84}NiGeN_3PSi$, $C_{32}H_{12}BF_{24}$	$C_{64}H_{96}CoGeN_3PSi$, $C_{32}H_{12}BF_{24}$
formula wt	1946.06	1962.25	1948.92	1961.02
crystal syst.	triclinic	monoclinic	monoclinic	monoclinic
space group	*P-1*	$P2_1$	$P2_1/n$	$P2_1/c$
a (Å)	13.166(3)	14.796(3)	24.597(5)	17.795(4)
b (Å)	17.038(3)	20.356(4)	15.214(3)	20.002(4)
c (Å)	23.353(5)	16.119(3)	25.476(5)	26.682(5)
α (deg.)	93.07(3)	90	90	90
β (δεγ)	99.64(3)	94.65(3)	102.25(3)	96.70(3)
γ (deg.)	92.76(3)	90	90	90
vol (Å^3)	5148.4(18)	4838.9(17)	9316(3)	9432(3)
Z	2	2	4	4
ρ(calc) (g.cm^{-3})	1.255	1.347	1.389	1.381
μ (mm^{-1})	0.546	0.603	0.650	0.642
F(000)	2002	2030	4012	4060
T (K)	150(2)	150(2)	150(2)	150(2)
reflns collect.	66978	66140	133090	131779
unique reflns	20136	18071	18296	18502
R_{int}	0.0325	0.0627	0.0752	0.1246
R1 [$I>2\sigma(I)$]	0.0545	0.0517	0.0541	0.0697
*w*R2 (all data)	0.1531	0.1236	0.1379	0.1929
CCDC No.	2513061	2513062	2513063	2513064

Responses to B-level alerts (also reported in CIFs):

Compound **2**:

PLAT910

PROBLEM: Missing FCF Reflection(s) Below Theta(Min)[Deg] = 2.20 Note

RESPONSE: Given the overall quality and completeness of the data (R1 = 5.45%; completeness = 99.5%), this does not affect the accuracy of the structure.

Compound **4a**:

PLAT910

PROBLEM: Missing FCF Reflection(s) Below Theta(Min)[Deg]= 2.40 Note

RESPONSE: Given the overall quality and completeness of the data (R1 = 5.41%; completeness = 99.9%), this does not affect the accuracy of the structure.

Compound **4b**:

PLAT221

PROBLEM: Solv./Anion Resd 2 F Ueq(max)/Ueq(min) Range 10.0 Ratio

RESPONSE: This is due to disorder in the BArF anion, which has been modelled.

PLAT910

PROBLEM: Missing FCF Reflection(s) Below Theta(Min)[Deg]= 2.52 Note

RESPONSE: Given the overall quality and completeness of the data (R1 = 6.97%; completeness = 99.9%), this does not affect the accuracy of the structure.

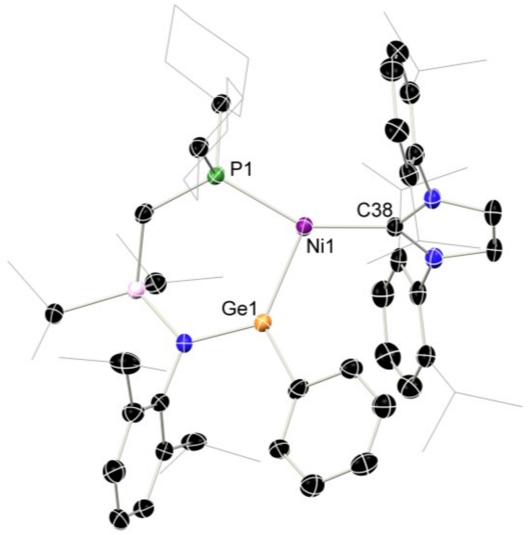

Figure S36. The molecular structure of **4b**, with thermal elipsoids at 30% probability, and hydrogen atoms removed for clarity.

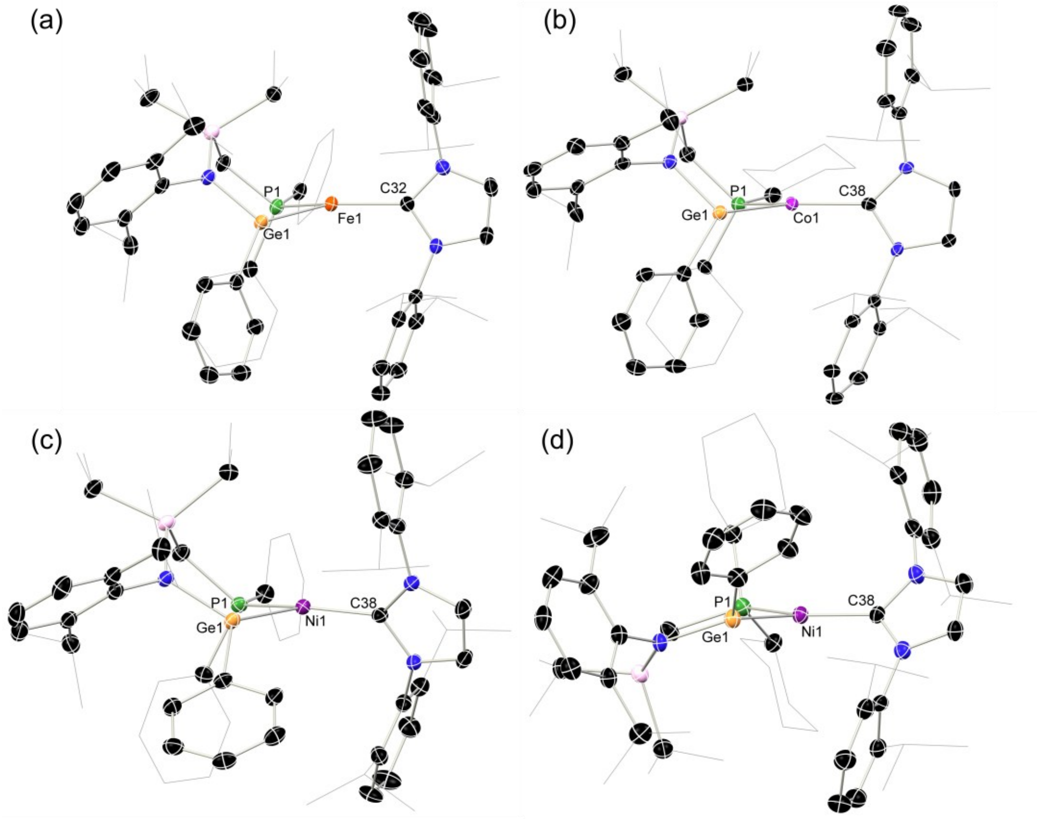

Figure S37. Side-on view of compounds (a) **2**, (b) **3b**, (c) **4a**, and (d) **4b**, showing the degrees of boat-like conformation through C-H anagostic interactions with M (M = Fe, Co, Ni).

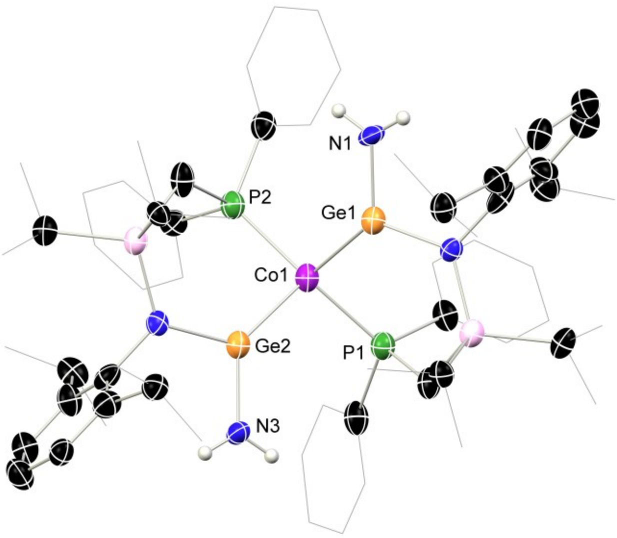

Figure S38. Molecular structure of $\{^{Ph}L(NH_2)Ge\}_2Co$, with thermal ellpsoids at 30%, and hydrogen atoms omitted aside from those on Ge-NH_2 ligands. Full data is not reported given its poor quality. This is presented only to show connectivity.

3. Computational methods and details

Computational experiments were performed using the ORCA 5.0.4 program, on the cationic parts of complexes described in the main text.[9] This utilised truncated models in which ^{i}Pr and P-*Ph* groups are replaced with methyl substituents (see Fig. S35 in Supporting Information), due to the size of the real structures leading to non-feasible computation times for CAS-SCF calculations. Initial DFT optimization of hydrogen positions, with heavy-atom position locked, was carried out at the ωB97XD level with the def2-SVP basis set for all atoms.[10],[11],[12],[13] The RIJCOSX approximation was also used throughout.[14] Molecular coordinates are included as a separate file.

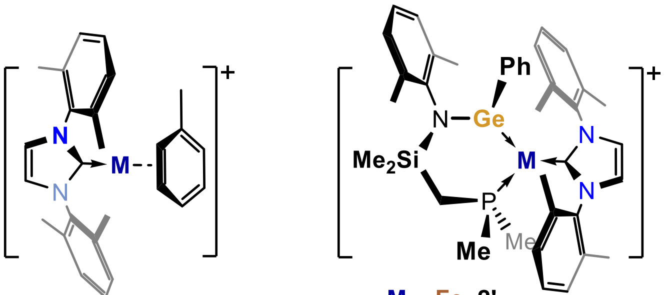

[IXyl·**M**(η^6-tol)]
M = **Fe**, **Co**, **Ni**)

M = **Fe**; **2'**
M = **Co**; **3'**
M = **Ni**; **4'**

Figure S39. Side-on view of compounds (a) **2**, (b) **3b**, (c) **4a**, and (d) **4b**, showing the degrees of boat-like conformation through C-H anagostic interactions with M (M = Fe, Co, Ni).

CAS-SCF calculations were carried out using DFT orbitals as an initial guess. This involved first fragmenting the complexes into the respective metal atom, and remaining ligand fragments, and recombining output .gbw files using the ORCA 'MergeFrag' program. This merged .gbw is used as the initial guess in each case. For all [IXyl·M(η^6-tol)]$^+$ complexes the active space comprises the five d-orbitals; for **2'-4'**, the vacant Ge-centred *p*-orbital is included, with the following work-flow: the initial CAS-SCF calculations for complexes **2'-4'** utilised active spaces consisting of the d-orbitals (*i.e.* 7,5, 8,5 and 9,5). The active space was expanded through a search for partner orbitals (PMOs), using the '*intorb pmos*' and '*extorb pmos*' keywords. In all cases, this identified only the Ge-centred vacant p-orbital as a partner, which was subsequently included in the active space. Interactions with other ligand orbitals were not found. Given the high-spin nature or otherwise (partially) filled *d*-orbitals in all systems, the lone electron pair at Ge is presumed to coordinate the *d*-block metal's vacant *s*-orbital. These two orbitals are therefore not included in the active space in these complexes. Thus, for [IXyl·M(η^6-tol)]$^+$ species active spaces of (7,5) (Fe), (8,5) (Co), and (9,5) (Ni) are employed, and

(7,6), (8,6), and (9,6) for complexes **2'-4'**, respectively. For these latter complexes, the 6th orbital is the vacant *p*-orbital at Ge. Where ground-state near degeneracy/low-lying excited states were observed (*i.e.* roots within ~0.25 eV of the 0th root), the additional roots were included in the final ground-state calculation. In all cases, the final calculation included n-electron valence state perturbation theory (NEVPT2) corrections, yielding the final reported results. A summary of contributions to the wavefuntions for all 6 calculated species if given in Tables S2-S5.

Table S2. Summary of the contributions to the wavefunctions of all [IXyl·M(η^6-tol)]$^+$ species, with NEVPT2 correction.

Root number	Relative energy (eV)	Relative energy (cm^{-1})	Electronic state	
		**Fe [CAS(7,5)]**		
0	0.000	0.0	0.63929	22111
			0.35962	21211
1	0.068	546.0	0.63912	21211
			0.3599	22111
		**Co [CAS(8,5)]**		
0	0.000	0.0	0.95859	22211
			0.04141	21122
		**Ni [CAS(9,5)]**		
0	0.000	0.0	0.99537	22221
			0.00463	22212
1	0.197	1587.9	0.99537	22212
			0.00463	22221

Table S3. Summary of the contributions to the wavefunction in **2'**, with NEVPT2 correction.

Root number	Relative energy (eV)	Relative energy (cm^{-1})	Electronic state	
		**Fe [CAS(7,6)]**		
0	0.000	0.0	0.35181	211210
			0.31011	212110
			0.08016	111211
			0.07144	112111
			0.0677	11212
			0.06094	12112
			0.03478	221110
			0.00773	121111
			0.00672	21112
1	0.018	144.9	0.41263	221110
			0.22656	212110

			0.0919	121111
			0.07844	21112
			0.06241	211210
			0.05207	112111
			0.04451	12112
			0.0141	111211
			0.01219	11212
			0.28054	211210
			0.25494	221110
			0.15551	212110
			0.06726	111211
2	0.108	869.1	0.059	121111
			0.05478	11212
			0.04905	21112
			0.03778	112111
			0.03141	12112
			0.00281	121210

Table S4. Summary of the contributions to the wavefunction in **3'**, with NEVPT2 correction.

Root number	Relative energy (eV)	Relative energy (cm^{-1})	Electronic state	
**Co [CAS(8,6)]**				
			0.58291	222110
			0.19183	221210
			0.08082	202112
			0.05818	212111
			0.02683	201212
0	0.000	0.0	0.01816	211211
			0.00759	121121
			0.00547	212210
			0.00506	121211
			0.0033	121220
			0.00304	122120
			0.00256	111122
			0.57741	221210
			0.19788	222110
			0.07833	201212
			0.05982	211211
1	0.215	1735.9	0.02643	202112
			0.02131	212111
			0.01437	122120
			0.00405	211112
			0.00351	122111

Table S5. Summary of the contributions to the wavefunction in **4'**, with NEVPT2 correction.

Root number	Relative energy (eV)	Relative energy (cm^{-1})	Electronic state	
**Ni [CAS(9,6)]**				
0	0.000	0.0	0.73154	222210
			0.1665	222012
			0.09622	222111
			0.00315	211221

Ab-initio ligand field theory (AILFT)

For complexes [IXyl·M(η^6-tol)]$^+$, additional AILFT calcualtions were carried out in order to further refine the energetic separation of the predicted *d*-orbitals. This was of particular interest given the apparent non-Aufbau electronic structure of the iron(I) derivative, which makes it a particularly interesting candidate for further more detailed study (*i.e.* in a later publication). This used the same functional and basis set as above (ωB97XD//def2-SVP); we include data for the CAS-SCF and CAS-SCF/NEVPT2 calculations. All possible excited states for the Fe^I (d^7), Co^I (d^8), and Ni^I (d^9) species were included, as outlined earlier by Neese *et al.*,[15] with the above utilised minimal active space (*i.e.* CAS(*n*,5); *n* = 7, 8, or 9). The resulting energies are given in Tables S6-S8, and suggest the non-Aufbau nature of the iron derivative.

Table S6. Results of the CAS-SCF AILFT calculation for [IXyl·**Fe**(η^6-tol)]$^+$.

	Orbital	Energy (eV)	Energy (cm^{-1})	Orbital character				
				d_{z2}	d_{xz}	d_{yz}	d_{x2-y2}	d_{xy}
w/o NEVPT2	1	0.000	0.0	0.005	-0.154	0.043	0.137	0.977
	2	0.027	217.5	0.060	0.069	0.167	0.973	-0.133
	3	0.550	4435.3	0.910	0.004	0.395	-0.125	-0.004
	4	0.913	7365.7	-0.395	-0.247	0.875	-0.117	-0.059
	5	0.940	7582.1	0.110	-0.954	-0.220	0.078	-0.153
w/ NEVPT2	1	0.000	0.0	0.004	-0.159	0.041	0.132	0.978
	2	0.040	325.5	0.061	0.066	0.173	0.973	-0.128
	3	0.514	4144.7	0.922	-0.005	0.368	-0.123	-0.003
	4	0.989	7976.1	-0.370	-0.256	0.882	-0.124	-0.060
	5	1.010	8145.7	0.098	-0.951	-0.235	0.080	-0.156

Table S7. Results of the CAS-SCF AILFT calculation for [IXyl·**Co**(η^6-tol)]$^+$.

	Orbital	Energy (eV)	Energy (cm^{-1})	Orbital character				
				d_{z2}	d_{xz}	d_{yz}	d_{x2-y2}	d_{xy}
w/o NEVPT2	1	0	0	-0.03	-0.39	0.208	0.326	0.837
	2	0.003	28.2	0.017	-0.23	-0.4	-0.828	0.316
	3	0.333	2683.4	0.614	-0.07	0.709	-0.333	-0.06

	4	0.751	6053.7	0.005	-0.89	-0.08	0.119	-0.44
	5	0.889	7173.9	0.789	0.054	-0.54	0.288	0.073
**w/ NEVPT2**	1	0	0	0.033	0.329	-0.29	-0.494	-0.75
	2	0.003	27.3	-0.01	0.306	0.351	0.739	-0.49
	3	0.403	3247.5	0.62	-0.08	0.704	-0.332	-0.06
	4	0.94	7582.1	0.006	-0.89	-0.08	0.12	-0.44
	5	1.094	8825	0.784	0.058	-0.54	0.292	0.075

Table S8. Results of the CAS-SCF AILFT calculation for [IXyl·**Ni**(η^6-tol)]$^+$.

	Orbital	Energy (eV)	Energy (cm^{-1})	Orbital character				
				d_{z2}	d_{xz}	d_{yz}	d_{x2-y2}	d_{xy}
**w/o NEVPT2**	1	0.000	0.0	0.070	0.365	-0.261	0.125	0.882
	2	0.004	36.0	-0.007	0.182	0.401	-0.882	0.169
	3	0.188	1518.1	0.685	0.182	-0.588	-0.286	-0.264
	4	0.504	4066.9	0.215	0.771	0.448	0.313	-0.248
	5	0.665	5361.5	0.692	-0.454	0.474	0.164	0.250
**w/ NEVPT2**	1	0.000	0.0	0.070	0.365	-0.261	0.125	0.882
	2	0.009	73.0	0.007	-0.182	-0.401	0.882	-0.169
	3	0.286	2309.1	-0.685	-0.182	0.588	0.286	0.264
	4	0.784	6326.6	-0.215	-0.771	-0.448	-0.313	0.248
	5	0.970	7823.5	-0.692	0.454	-0.474	-0.164	-0.250

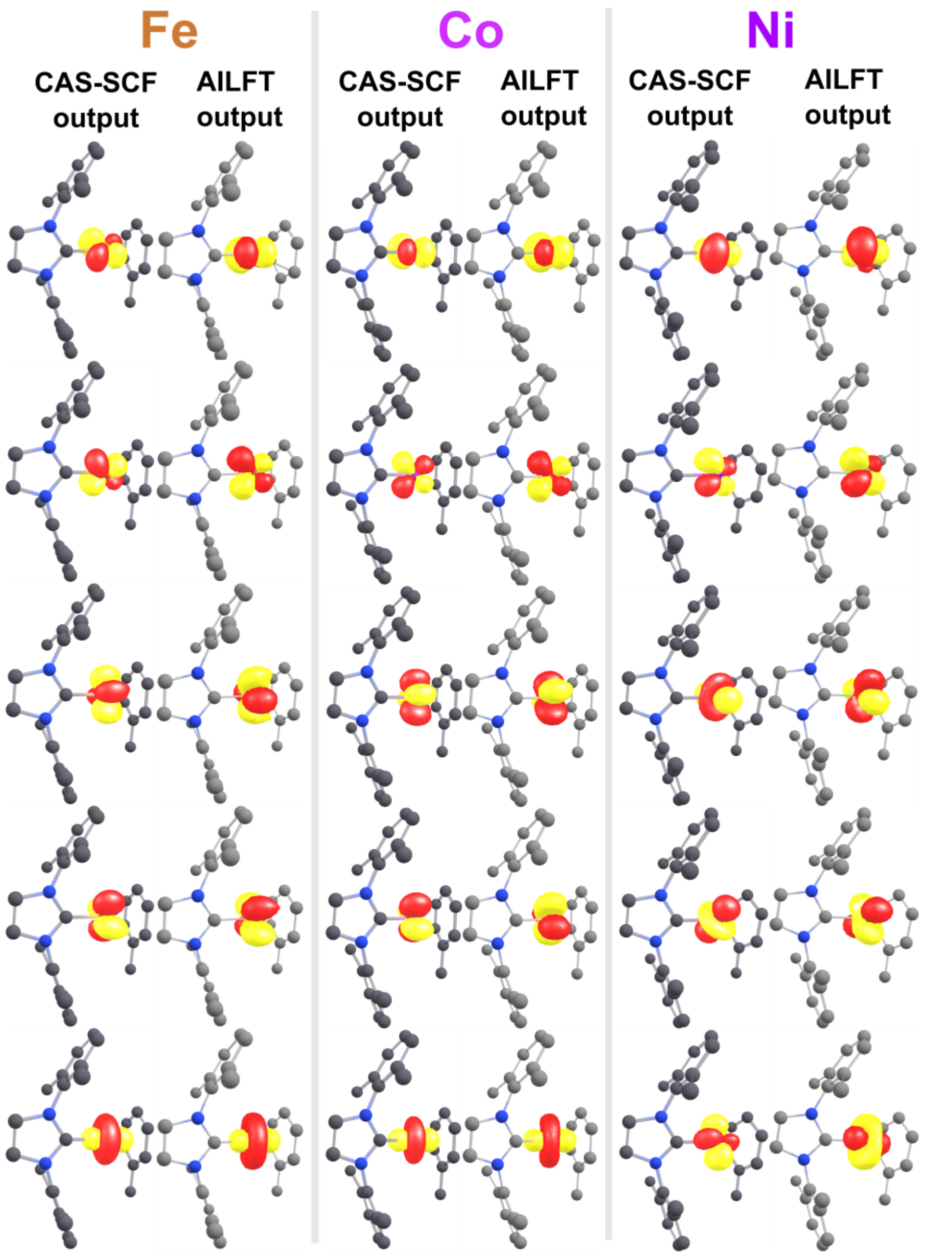

Figure S40. Comparison of active space output orbitals from CAS-SCF and AILFT for [IXyl·M(η^6-tol)]$^+$ species. High similarity demonstrates the high *d*-character of the

4. References

[1] A. Schulz, T. L. Kalkuhl, P. M. Keil, T. J. Hadlington, “T-shaped Ni0 Systems Featuring Cationic Tetrylenes: Direct Observation of L/Z-type Ligand Duality, and Alkene Hydrogenation Catalysis” *Angewandte Chemie International Edition* **2023**, *62*, e202305996.

[2] S. C. Bart, E. Lobkovsky, P. J. Chirik, “Preparation and Molecular and Electronic Structures of Iron(0) Dinitrogen and Silane Complexes and Their Application to Catalytic Hydrogenation and Hydrosilation” *J. Am. Chem. Soc.* **2004**, *126*, 13794–13807.

[3] P. J. Chirik, “Iron- and Cobalt-Catalyzed Alkene Hydrogenation: Catalysis with Both Redox-Active and Strong Field Ligands” *Acc. Chem. Res.* **2015**, *48*, 1687–1695.

[4] Y. Gao, L. Wang, L. Deng, “Distinct Catalytic Performance of Cobalt(I)– *N* -Heterocyclic Carbene Complexes in Promoting the Reaction of Alkene with Diphenylsilane: Selective 2,1-Hydrosilylation, 1,2-Hydrosilylation, and Hydrogenation of Alkene” *ACS Catal.* **2018**, *8*, 9637–9646.

[5] K. Tokmic, C. R. Markus, L. Zhu, A. R. Fout, “Well-Defined Cobalt(I) Dihydrogen Catalyst: Experimental Evidence for a Co(I)/Co(III) Redox Process in Olefin Hydrogenation” *J. Am. Chem. Soc.* **2016**, *138*, 11907–11913.

[6] **N.d.**

[7] G. M. Sheldrick, “*SHELXT* – Integrated space-group and crystal-structure determination” *Acta Crystallogr A Found Adv* **2015**, *71*, 3–8.

[8] G. M. Sheldrick, “Crystal structure refinement with *SHELXL*” *Acta Crystallogr C Struct Chem* **2015**, *71*, 3–8.

[9] F. Neese, “The ORCA program system” *WIREs Comput Mol Sci* **2012**, *2*, 73–78.

[10] A. D. Becke, “Density-functional thermochemistry. V. Systematic optimization of exchange-correlation functionals” *The Journal of Chemical Physics* **1997**, *107*, 8554–8560.

[11] F. Weigend, R. Ahlrichs, “Balanced basis sets of split valence, triple zeta valence and quadruple zeta valence quality for H to Rn: Design and assessment of accuracy” *Phys. Chem. Chem. Phys.* **2005**, *7*, 3297.

[12] J.-D. Chai, M. Head-Gordon, “Systematic optimization of long-range corrected hybrid density functionals” *The Journal of Chemical Physics* **2008**, *128*, 084106.

[13] S. Grimme, J. Antony, S. Ehrlich, H. Krieg, “A consistent and accurate ab initio parametrization of density functional dispersion correction (DFT-D) for the 94 elements H-Pu” *The Journal of Chemical Physics* **2010**, *132*, 154104.

[14] F. Neese, F. Wennmohs, A. Hansen, U. Becker, “Efficient, approximate and parallel Hartree–Fock and hybrid DFT calculations. A ‘chain-of-spheres’ algorithm for the Hartree–Fock exchange” *Chemical Physics* **2009**, *356*, 98–109.

[15] S. K. Singh, J. Eng, M. Atanasov, F. Neese, “Covalency and chemical bonding in transition metal complexes: An ab initio based ligand field perspective” *Coordination Chemistry Reviews* **2017**, *344*, 2–25.
